# Infectious bursal disease virus replication is inhibited by avain T cell chemoattractant chemokine CCL19

**DOI:** 10.3389/fmicb.2022.912908

**Published:** 2022-07-22

**Authors:** Qiuxia Wang, Fuming Chu, Xin Zhang, Huilong Hu, Lang Lu, Fang Wang, Yan Yu, Yanhong Zhang, Jinyou Ma, Zhiyong Xu, Fatma Eldemery, Changbo Ou, Xingyou Liu

**Affiliations:** ^1^College of Animal Science and Veterinary Medicine, Henan Institute of Science and Technology, Xinxiang, China; ^2^Department of Hygiene and Zoonoses, Faculty of Veterinary Medicine, Mansoura University, Mansoura, Egypt; ^3^College of Animal Science and Technology, Guangxi University, Nanning, China; ^4^College of Life Science, Xinxiang University, Xinxiang, China

**Keywords:** CCL19, infectious bursal disease virus, virus replication, T cell, chemokine

## Abstract

Chemokine CCL19, together with its receptor CCR7, is one of the most important factors recruiting immune cells into target organ during virus infection. Our previous study has shown that CCL19 played a vital role in the process of T cell trafficking into bursae during bursal disease virus (IBDV) infection. In this study, we hypothesized that CCL19 could exert direct influences on IBDV replication other than recruiting immune cells. A eukaryotic expression vector of pEGFP-N1/CCL19 was successfully constructed and identified by PCR, double enzymes digestion, and sequencing. Different concentrations of pEGFP-N1/CCL19 plasmids were transfected into DF1 cells and CCL19 protein was highly expressed. Then, DF1 cells were infected with IBDV B87 strain post-transfection. Based on PCR and Western blot results, CCL19 could obviously decrease the gene levels of VP1 and VP2 and the protein levels of VP2 and VP3. When CCL19 was knocked down, the gene levels of VP1 and VP2 were significantly upregulated. Moreover, indirect immunostaining revealed that the IBDV content was largely decreased after CCL19 overexpression. Additionally, CCL19 inhibitory effects might rely on activation of the JNK signal pathway. Taken together, chemokine CCL19 directly blocks IBDV replication in DF1 cells, indicating that CCL19 could play crucial functions other than recruiting T cells during the pathogenesis of IBDV.

## Introduction

Infectious bursal disease virus (IBDV) is one of the most economically important viruses affecting poultry industry worldwide. It induces acute, highly contagious immunosuppressive diseases in young chickens aged 3 to 15 weeks (Sharma et al., 2000; Eterradossi and Saif, 2013). IBDV is a non-enveloped Avibirnavirus, which belongs to the family Birnaviridae that group viruses enclosing bisegmented (segments A and B) double-stranded RNA genomes (Eterradossi and Saif, 2013; He et al., 2021). Genome segment B encodes viral protein VP1, an RNA-dependent RNA polymerase. Genome segment A contains two partially overlapping open reading frames that encode a non-structural polypeptide, known as VP5, and a large precursor polyprotein, which is further cleaved into VP2 (outer capsid), VP3 (inner capsid), and VP4 (a serine protease). VP2 and VP3 are the major structural proteins, comprising 51 and 40% of the virion, respectively, that have a crucial role in the morphogenesis and encapsulation of the viral genome (Berg, 2000; Luque et al., 2009).

Following IBDV infection in chicken, there was a dramatic infiltration of T cells into the bursa of Fabricius, and was first detectable as early as 1-day post-infection and persisted for several weeks (Tanimura and Sharma, 1997; Kim et al., 2000; Sharma et al., 2000). It has been indicated that the highest number of bursal cells were T cells (up to 65% of lymphocytes) (Kim et al., 2000; Sharma et al., 2000). The number of infiltrating T cells have been shown to be correlated with the IBDV virulence; however, the way in which the T cells inhibited the IBDV infection was different (Tippenhauer et al., 2013; Yu et al., 2015), and that may be due to the inconclusive differentiation of T cells into bursa of Fabricius. Ruan et al. believed that the infiltrating T lymphocytes in bursa of Fabricius were mainly CD4^+^T cells and CD4^+^CD8^+^T cells after IBDV infection (Ruan et al., 2020). The study of Dobner et al. showed that the infiltrating T cells reached the maximum in the bursa of Fabricius on the 7th day after IBDV infection, and the number of CD8^+^T cells was more than that of CD4^+^T cells; CD4^+^T cells increased by 4–11 times, CD8^+^T cells increased by 11–38 times, and then gradually decreased, and the decline rate of CD8^+^T cells was more moderate (Dobner et al., 2020). This may be due to differences in virulence of the virus used for infection or in the genotype of the chicken. Previous reports have demonstrated various biological functions of the infiltrating bursal T cells, such as promoting viral clearance, tissue damage and delaying follicular recovery, early immunopathology during an IBDV infection, and contribution to B cell genomic instability (Kim et al., 2000; Rautenschlein et al., 2002; Yu et al., 2015; Ciccone et al., 2017).

CC chemokine ligand 19 (CCL19), also known as macrophage inflammatory protein-3β and EBI-1 ligand chemokine, always, together with its chemokine receptor CCR7, plays a pivotal role in T cell and dendritic cell trafficking into lymphoid tissue (Forster et al., 2008). Previous studies indicated that chemokines, especially CCL19, played an important role in T cell migration into bursae during IBDV infection (Ou et al., 2017a,b; Wang et al., 2019). The mRNA expressions of CCL19 were largely increased on day 1 and day 3 post-IBDV infection, and it could interact with many differentially expressed genes after IBDV infection (Ou et al., 2017b). Moreover, our previous study showed that, during the process of T cell migration into bursae of Fabricius, the axis of CCR7/CCL19 was significantly elevated and the chemokine CCL19 acts as a chicken chemotactic factor facilitating the infiltration of T cells into the bursae in IBDV infection (Wang et al., 2019). Although the role of chemokines is recruiting immune cells to the site of virus infection, the chemokines could directly regulate the virus replication. For instance, five chemokines have been identified to contribute to the control of human immunodeficiency virus (HIV) replication. In HIV-infected individuals, the combination of the five chemokines (CCL14, CCL21, CCL27, XCL1, and SDF-1BETA) was found to upregulate the activation markers CD69 and CCR6, and downregulate the key HIV coreceptors CXCR4 and CCR7 on CD4+ T cells. Meanwhile, the anti-HIV host restriction factors IFITM1 and IFITM2 were significantly expressed, resulting in HIV replication reduction (Jacobs et al., 2017). In addition, Yu et al. provided insights for the roles of cytokines in porcine epidemic diarrhea virus (PEDV) replication and demonstrated that overexpression of CCL2, CCL5, and CCL8 significantly inhibited the virus replication, but silencing of these chemokine genes significantly promoted PEDV replication (Yu et al., 2019). Yan et al. found that CCL19 could rapidly clear HBV in the liver of mice by enhancing the responses of CD8^+^T cells (Yan et al., 2021). Goto et al. demonstrated that human CAR-T cells producing human IL-7 and CCL19 can generate the potent therapeutic efficacy against solid tumors (Goto et al., 2021). But the effects of cytokines and chemokines on IBDV replication are still unclear.

Therefore, in this study, we aimed to investigate whether CCL19 could regulate the IBDV replication. The role of CCL19 on IBDV replication was evaluated *in vitro* through over-expression and knockdown of CCL19. CCL19 has further biological effects during the process of IBDV pathogenesis.

## Materials and methods

### Cells and virus

Chicken fibroblast cell line DF1 cells (ATCC CRL-12203) were cultured in Dulbecco's modified eagle's medium (DMEM) with 10% fetal bovine serum (Gibco, USA). After DF1 cells were split for transfection, the cells were maintained in 2% fetal bovine serum. Intermediate virulence vaccine strain IBDV B87 was purchased from Vland Biotech Group (Qingdao, China) and amplified in DF1 cells.

### Reagents and antibodies

The JNK inhibitor, p38 inhibitor, Akt inhibitor, and Erk inhibitor were purchased (MedchemExpress, Shanghai, China). DF1 cells were treated with either dimethyl sulfoxide (DMSO), which is the solvent for these four inhibitors, or 20 μM of each of these four inhibitors for 2 h before infection at 37°C in 5% CO_2_ incubator. Then, the cells were inoculated with IBDV B87 virus at a multiplicity of infection (MOI) of 10 TCID_50_ at 37°C in 5% CO_2_ incubator. After 1 h of virus adsorption, the virus medium was removed and fresh medium containing fresh inhibitor was added into the culture.

Mouse antibodies against VP2 and VP3 were purchased from Wuhan Biorbyt. Rabbit antibodies against JNK and p-JNK were purchased from Cell Signaling. Mouse antibody against GAPDH and horseradish peroxidase (HRP)-labeled goat antibody against mouse and rabbit IgG were purchased from Nanjing Bioworld. Cy3-conjugated goat anti-mouse IgG antibody (1:100) was purchased from Wuhan Boster.

### Construction of eukaryotic expression vector PEGFP-N1/CCL19

Previously constructed pMD T/CCL19 plasmid was used as the template for amplifying CCL19 gene (Wang et al., 2018). A pair of primers with restriction enzyme sites *Hind* III and *BamH* I was designed to amplify CCL19 by polymerase chain reaction (PCR). The purified CCL19 fragment was inserted into empty vector pEGFP-N1 by using double enzymes digestion method to generate pEGFP-N1/CCL19. Then, the plasmid was transformed into competent cells DH5α. The positive clones were selected, purified using plasmid extraction kit (Omega), and confirmed by PCR, double enzymes *Hind* III and *BamH* I digestion, and sequenced by a commercial company (Sangon Biotech, Shanghai, China).

### CCL19 knockdown by siRNA

Three siRNA oligos segments (si817, si717, and si582) as well as the NT siRNA were designed and synthesized by Genepharma (Shanghai, China). The DF1 cells were seeded in six-well culture plates (Corning Inc., USA) at a density of 2 × 10^5^ cells/well in six-well plates and incubated for 24 h before transfection at 37°C in 5% CO_2_ incubator. Then, siRNA into CCL19 and NT siRNA were transfected into the cells mediated by Lipofectamine2000^TM^, respectively. At 48 h after transfection, total RNA was extracted and reversely transcribed into cDNA to detect the inhibitory efficiency of siRNA through real-time PCR (qRT-PCR).

### Transfection and virus challenge

The DF1 cells seeded in six-well culture plates (Corning Inc., USA) using DMEM medium and supplemented with 8% fetal bovine serum (Gibco, USA) were transfected with different concentrations (0.5, 1.0, 1.5, and 2.0 μg) of pEGFP-N1/CCL19 plasmids using Lipofectamine^TM^ 2000 (Invitrogen, Grand Island, USA) following the manufacturer's protocols. Meanwhile, mock DF1 cells transfected with empty plasmid pEGFP-N1 were used as a control. At 24 h post-transfection, DF1 cells were gently washed with serum-free DMEM medium and challenged with 1 TCID_50_ of IBDV B87 strain and incubated at 37 °C in a 5% CO_2_ incubator. Cells were collected at 24, 36, 48, 60, and 72 h after infection.

### Cell viability assay

Cell viability in DF-1 cells was measured by the Cell Counting Kit-8 (CCK-8) assay kit (Solarbio, Beijing, China). Briefly, DF-1 cells were seeded at a density of 2 × 10^5^ cells/ml in 96-well culture plates (100 μl/well) in media, and then, the culture plates were incubated in 5% CO_2_ atmosphere incubator at 37°C 24 h before transfection with pEGFP-N1-CCL19 at different concentrations. At 4 h after transfection, medium was replaced with 2% serum maintenance solution and incubated for 24 h; then, the medium was changed with serum-free media. Notably, 10 μl CCK-8 was added to each well and incubated for 1 h at 37°C.The optical density (OD) was measured at 452 nm. The percentage of viable cells was determined by the formula: ratio (%) = [OD (CCL19)–OD (Blank)]/[OD (Control)–OD (Blank)] × 100%.

### Real-time polymerase chain reaction (qRT-PCR)

To quantify the copy number of IBDV and type I interferons, the IBDV VP1 and VP2, IFN-α and IFN-β, interferon regulatory factor 7 (IRF7) genes were selected for qRT-PCR with β-actin as the housekeeping gene (Staines et al., 2016). Total RNA was extracted from collected DF1 cells using Tissue RNA extraction kit (Omega^TM^, Beijing, China) and reversely transcribed into cDNA with M-MLV reverse transcriptase (Promega, CA, USA) in accordance with the manufacturer's instructions. All primers used for the qRT-PCR are listed in Table 1. The qRT-PCR was carried out using SYBR Green PCR Master Mix (Invitrogen, Shanghai, China). The PCR conditions were as follows: 30 s at 95°C for initial denaturation, followed by 40 cycles at 95°C for 5 s, 58°C for 20 s, and 72°C for 20s. The data were analyzed using the threshold cycle (CT) values and gene expression calculated using the 2^−ΔΔCt^ method. All reactions were performed in three replicates to ensure the reproducibility of the amplification.

**Table 1:** Primers used for RT-PCR amplications.

**Primer name**	**Sequence (5'-3')**
CCL19-N1-F	CCGAAGCTT GCCACCATGCAGCGGCTGCACGTT
CCL19-N1-R	CCGGGATCCCTAATTGCCTTGATTTGGGAC
VP1-F	GAGGCGTTGAGGTTGGTA
VP1-R	ACTCAGTCCGGCTTCGTT
VP2-F	GGAGCCTTCTGATGCCAACAAC
VP2-R	CAGGAGCATCTGATCGAACTTGTAG
β-Actin-F	TTGTGATGGACTCTGGTGATGGTG
β-Actin-R	TTCTCTCTCGGCTGTGGTGGTG

*Underlined bold letters indicated the Hind III and BamH I restriction enzyme sites*.

### Western blotting

The DF1 cells were harvested at 24, 48, and 72 h post-IBDV infection and washed twice with pre-cold phosphate-buffered saline (PBS). The cells were lysed with RIPA Lysis Buffer (Solarbio, China) with 1% PMSF proteinase inhibitor (Sigma, USA) and the total protein was collected following the protocol instructions. The collected protein was separated using 12% SDS-PAGE and then blotted onto nitrocellulose membranes. Subsequently, the membranes were blocked in 5% non-fat milk at 37 °C for 2 h. After washing with PBS, the membranes were incubated with primary antibody, including mouse anti-VP2 and anti-VP3 (1:1,000) and anti-GAPDH antibody (1:5,000) at 4°C for 12 h. The membranes were washed three times with PBS, and incubated for 1 h at 37°C with horseradish peroxidase (HRP)-labeled goat anti-mouse IgG (1:5,000). After being washed four times with PBS, the proteins on the membranes were visualized using the Chemiluminescent ECL western blotting substrate (Solarbio, Beijing, China). Mock-infected DF1 cells were used as the negative control.

### Indirect immunostaining

DF1 cells collected at 24, 48, and 72 h post-infection were rinsed gently with PBS (pH 7.4), and fixed for 15 min at room temperature with 4% formaldehyde in PBS. Then, the cells were permeabilized with 1% Triton X-100 for 15 min at room temperature. The samples were blocked in 5% normal rabbit serum for 1 h. Furthermore, blocking solution was aspirated, and the samples incubated with mouse anti-VP2 antibody (1:1,000) for 1 h at 37°C. After removal of the primary antibody, the samples were incubated with Cy3-conjugated goat anti-mouse IgG secondary antibody (1:100) for 1 h at room temperature in a dark place. Then, the samples were analyzed using confocal microscopy immediately after being rinsed with PBS.

### Statistical analysis

All data were analyzed by one-way analysis of variance and the statistical significance between the negative control group and CCL19 infection group was indicated as follows: ^*^ (*p* < 0.05) and ^**^ (*p* < 0.01). All experiments were performed with three replicates, and the results were expressed as mean ± standard deviation (SD).

## Results

### IBDV B87 strain could grow in DF1 cells

**Figure 1:**
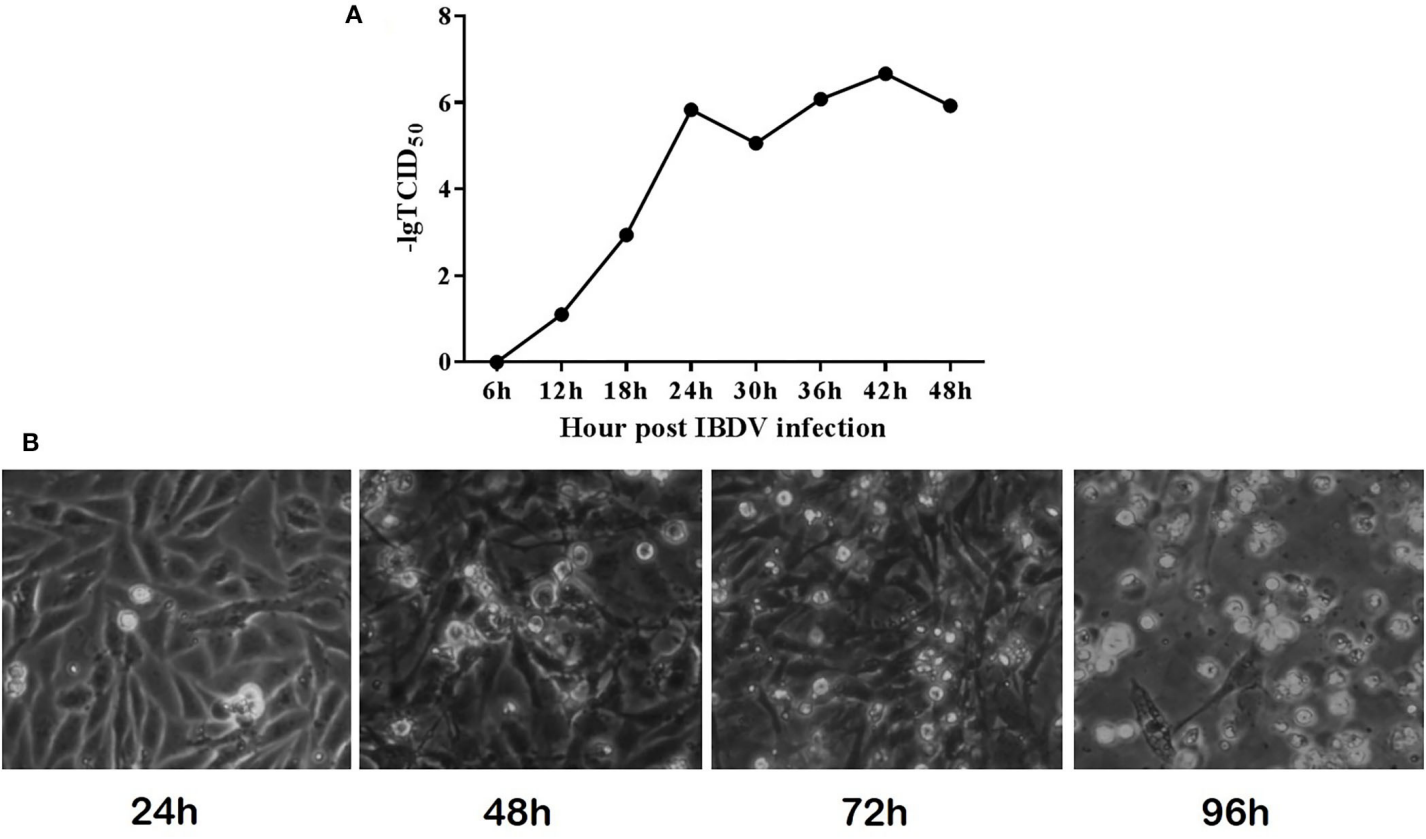
IBDV B87 strain could grow in DF-1 cells. **(A)** TCID_50_ of IBDV B87 strain in DF-1 cells. **(B)** Cell morphology at different time points after IBDV infection.

In order to determine the growth and replication of B87 in DF-1 cells, DF-1 cells were infected with different doses of virus, and TCID_50_ was detected and a growth curve was drawn. The results showed that IBDV B87 strain could replicate and proliferate in DF1 cells (Figure 1). With an increase in concentration and over time, B87 can cause marked pathological cell death.

### Identification and expression of PEGFP-N1/CCL19

**Figure 2:**
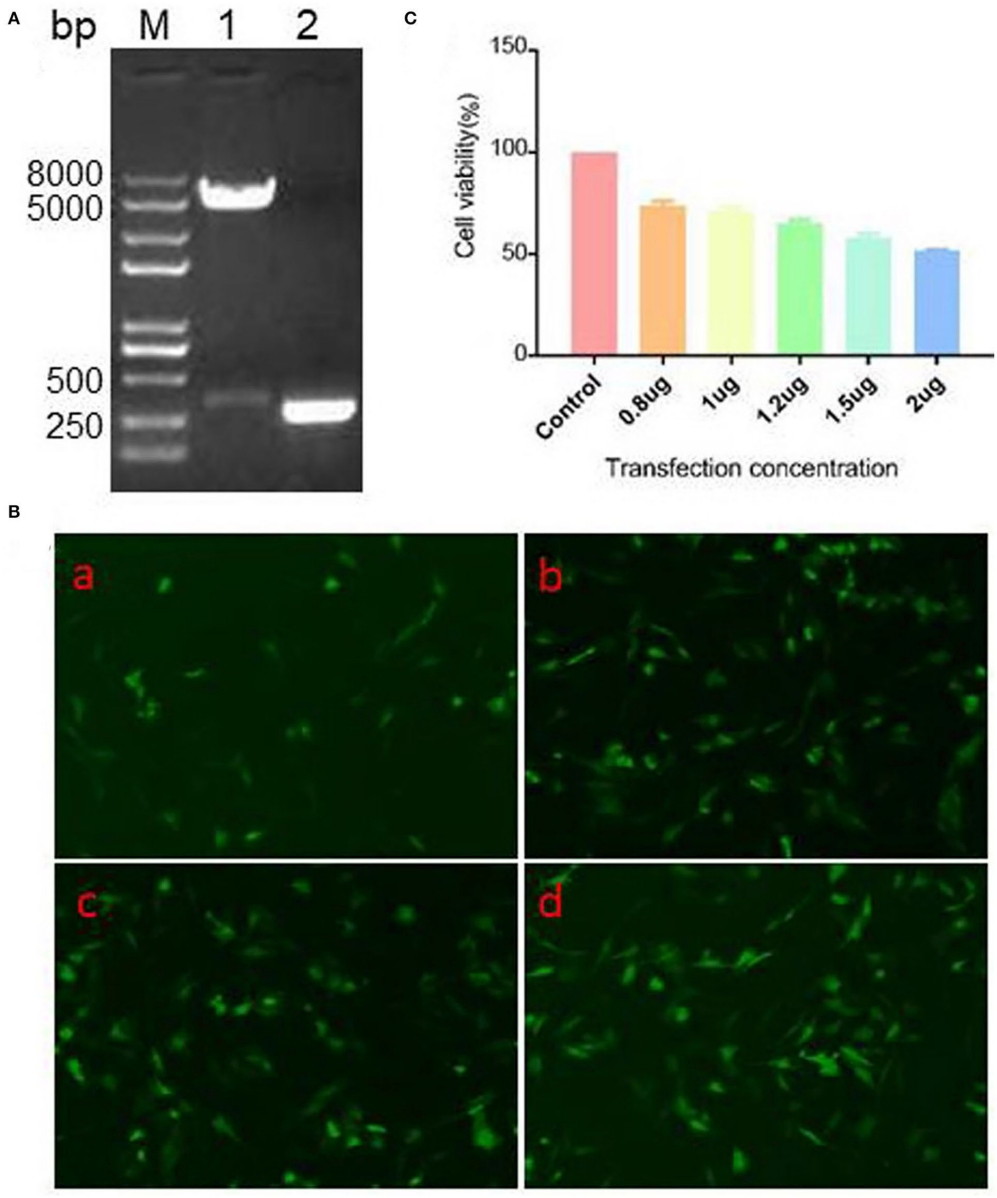
Identification, expression efficacy, and cell viability on DF1 cells of pEGFP-N1/CCL19. **(A)** PCR and double restriction enzyme digestion identification of eukaryotic expression vector pEGFP-N1/CCL19. Lane M: Trans 2K Plus II DNA marker; Lane 1: double enzymes digestion; Lane 2: PCR of CCL19. **(B)** Different concentrations (0.5, 1.0, 1.5, and 2.0 μg) of plasmid pEGFP-N1/CCL19 in DF1 cells were transfected into DF1 cells in **(B)** (a–d), respectively. **(C)** Effects of different concentrations of plasmid pEGFP-N1/CCL19 on cell viability. Concentrations of 0.8, 1.0, 1.2, 1.5, and 2.0 μg plasmids were transfected in DF1 cells.

After construction of pEGFP-N1/CCL19 plasmid, it was identified by double enzyme digestion and PCR (Figure 2A). As seen in Figure 2A, plasmid digestion with *Hind* III and *Bam*H I showed two bands. Moreover, there was a 300 bp target band after amplification with the specific primers of CCL19 (Figure 2A). Also, the plasmid was confirmed by sequencing through a commercial sequencing company (data not shown). All results indicated that the CCL19 was correctly inserted into the pEGFP-N1 plasmids.

To further confirm the expression efficacy of the plasmids in DF1 cells, different concentrations of pEGFP-N1/CCL19 plasmids were transfected into DF1 cells. At 24 h post-transfection, all tested concentrations of plasmids showed efficacy expression in DF1 cells (Figure 2B).

### Effect of different concentration PEGFP-N1/CCL19 on the cell viability of DF-1 cells

Before evaluating the role of CCL19 on IBDV infection, we observed the effect of different concentrations of pEGFP-N1/CCL19 on DF-1 cells viability by CCK-8 assay. Notably, concentrations of 0.8, 1.0, 1.2, 1.5, and 2.0 μg pEGFP-N1/CCL19 were transfected into DF-1 cells, respectively. The result showed that pEGFP-N1/CCL19 plasmid had certain cytotoxicity to DF-1 cells, and with the increase of transfection concentration, the cytotoxicity increased. When the transfection concentration was greater than 1.2 μg, the cell viability was lower than 60% (Figure 2C). Hence, pEGFP-N1/CCL19 was used at concentrations of 1.0 μg in all subsequent experiments.

### Overexpressed CCL19 suppresses IBDV replication and release in DF-1 cells

**Figure 3:**
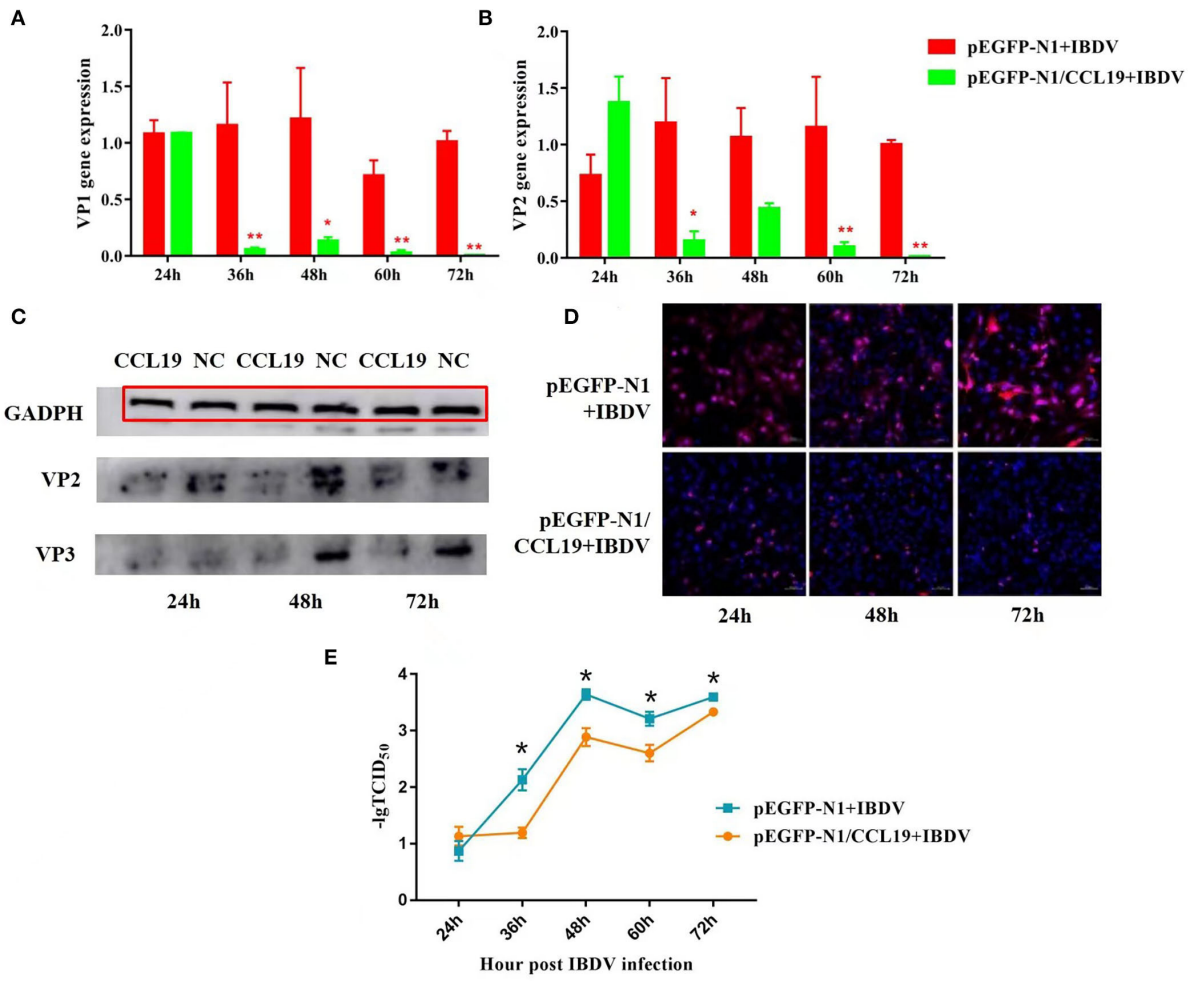
Negative regulation of overexpression CCL19 on IBDV replication at different time points in DF1 cells. Gene expression levels of VP1 **(A)** and VP2 **(B)** at 24, 36, 48, 60, and 72 h post-infection with IBDV B87 strain in pEGFP-N1/CCL19 group compared with empty vector plasmid pEGFP-N1 control group. **(C)** VP2 and VP3 protein expression levels of the empty vector plasmid pEGFP-N1 negative control group (NC) and pEGFP-N1/CCL19 group (CCL19) at 24, 48, and 72 h post-infection with IBDV. **(D)** Semi-quantification of IBDV by indirect immunofluorescent method of pEGFP-N1/CCL19 group compared with empty vector plasmid pEGFP-N1 negative control group at 24, 48, and 72 h post-infection with IBDV. Notably, 1.0 μg empty vector or pEGFP-N1/CCL19 plasmid was used in virus infection experiment. **(E)** TCID_50_ at 24, 36, 48, 60, and 72 h post-infection with IBDV B87 strain in pEGFP-N1/CCL19 group compared with empty vector plasmid pEGFP-N1 control group. * or ** indicates that there were significant differences of VP1, VP2 gene expression levels or TCID_50_ between pEGFP-N1 and pEGFP-N1/CCL19 groups (*p* < 0.05 or *p* < 0.01). The red box indicates the GAPDH band.

To test whether CCL19 can protect DF1 cells from IBDV infection, pEGFP-N1/CCL19 (1.0 μg) was transfected into DF1 cells before cells were infected with IBDV. Figure 3 demonstrates the direct effects of CCL19 overexpression in DF1 cells on IBDV replication.

Figures 3A,B display the VP1 and VP2 gene expression levels in IBDV-infected DF1 cells, respectively. At 24 h post-infection with IBDV, there were no significant differences in VP1 and VP2 gene expression levels between the pEGFP-N1/CCL19 group and the empty vector control group. However, from 36 h post-IBDV-infection, the pEGFP-N1/CCL19 plasmids strongly inhibit the gene expressions of both IBDV VP1 and VP2. These inhibitory effects lasted until the end of the experiment except at 48 h post-infection, where VP1 and VP2 genes displayed similar gene expression trends.

Moreover, the protein expression levels of VP2 and VP3 were determined by western blot (Figure 3C). As shown in Figure 3C, VP2 and VP3 protein levels were largely decreased by CCL19 at 24, 48, and 72 h post-IBDV-infection, which were consistent with the results of the gene expression levels. Then, the IBDV numbers were semi-quantified by indirect immunofluorescent method (Figure 3D). Obviously, there were more IBDV-positive cells in the empty vector negative control group and those of the pEGFP-N1/CCL19 group at 24, 48, and 72 h post-infection.

The effect of chemokine CCL19 on the release of IBDV virions was further investigated. DF-1 cells were seeded at a density of 2 × 10^5^ cells/well in six-well culture plates and incubated in 5% CO_2_ atmosphere incubator at 37°C 24 h before transfection with 1 μg pEGFP-N1-CCL19. Meanwhile, 1 μg empty vector was transfected as the control group. Then, cells were challenged with 1 TCID_50_ of IBDV B87 strain and incubated at 37°C in a 5% CO_2_ incubator. The cell supernatants were collected at 24, 36, 48, 60, and 72 h, respectively. TCID_50_ of the virus in the supernatant was detected (Figure 3E). The results showed that the virus titer in the PEGFP-N1-CCL19 transfected group was significantly lower than that in the control group from 36 h after IBDV infection.

### CCL19 knockdown promotes IBDV replication and release in DF-1 cells

**Figure 4:**
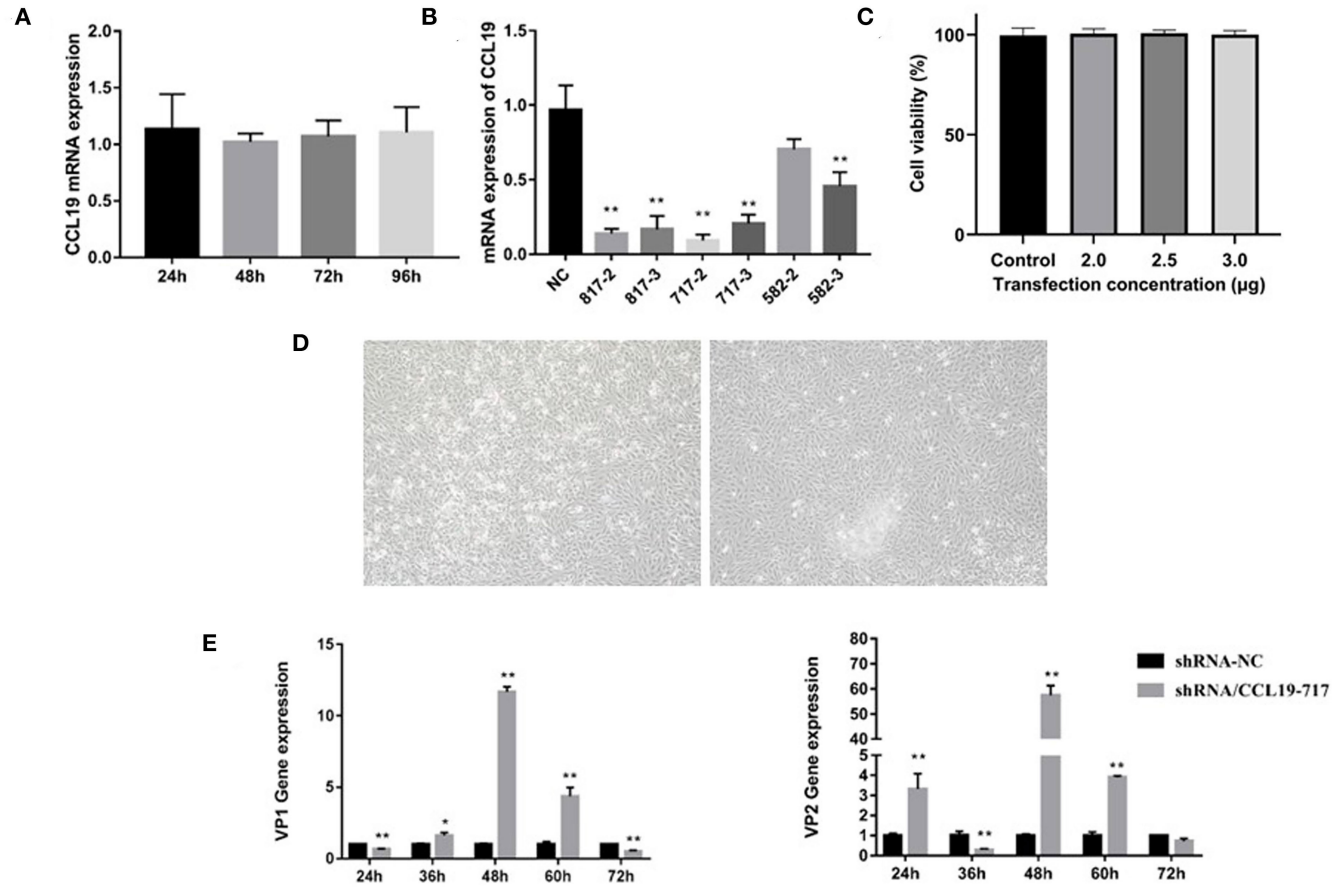
siRNA knockdown of CCL19 promotes IBDV replication. **(A)** Gene expression levels of CCL19 in DF1 cells. **(B)** The efficiency of different CCL19-siRNA. **(C)** Cell viability at 2 h after si717 transfection. **(D)** Cell morphology at 24 h after si717 transfection. **(E)** Gene expression levels of VP1 (left) and VP2 (right) at 24, 36, 48, 60, and 72 h post-infection with IBDV B87 strain in si717 group compared with NT control group. * or ** indicates that there were significant differences of VP1, VP2, and CCL19 gene expression levels between siCCL19 and NT groups (*p* < 0.05 or *p* < 0.01).

To further determine the role of CCL19 in IBDV infection, we first assessed the endogenous level of CCL19 in DF-1 cell lines (Figure 4A). We found that DF-1 cell exhibited general CCL19 expression levels. DF-1 cell lines were used for CCL19 knockdown using three different siRNAs (si817, si717, and si582). A subsequent qRT-PCR analysis showed that the CCL19 gene levels were reduced in the DF-1 cells transfected with si717 compared with the respective NT siRNA transfected control cells (Figure 4B). We selected si717 for further experiments; as the knockdown was more robust, qRT-PCR data showed that CCL19 mRNA levels were reduced by > 70% in DF-1 cell lines. Hence, DF-1 cells transfected with si717 were used in subsequent experiments.

Following siRNA transfection, we found that CCL19 knockdown slightly impacted DF-1 cell growth at 24 h (Figure 4C), although no significant change was observed in cell viability (Figure 4D). Indeed, IBDV VP1 and VP2 gene expression levels were increased obviously when there was a knockdown of CCL19 (Figure 4E). These findings indicate that the reduction of CCL19 expression may promote replication of IBDV in DF-1 cells.

### Blockage of JNK and p38 signal pathway inhibited the negative regulation of CCL19 on IBDV replication

**Figure 5:**
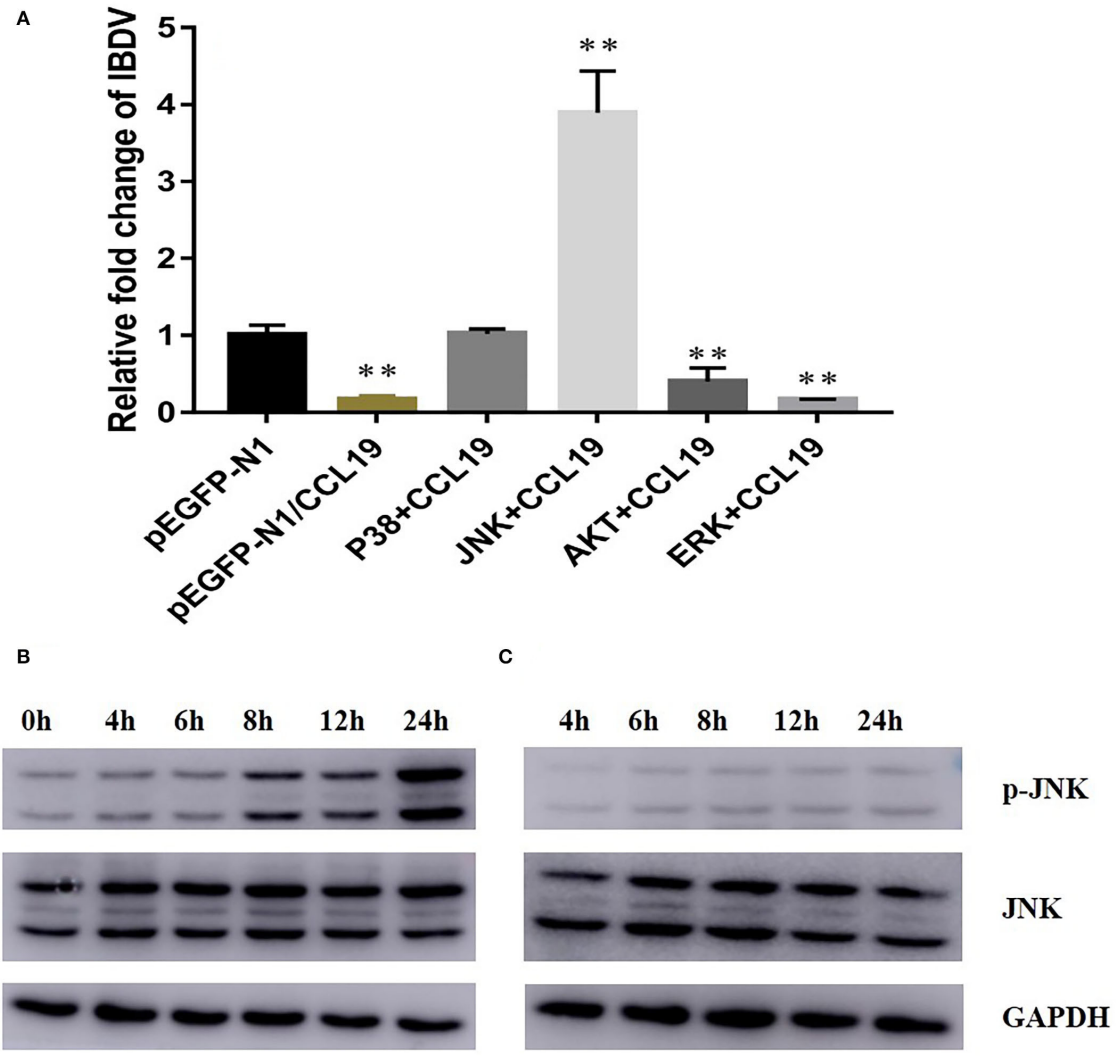
CCL19 activates JNK signal pathway to inhibit IBDV replication. **(A)** DF1 cells were pretreated with 20 μM of four signal pathway inhibitors before transfection with pEGFP-N1/CCL19 plasmid and then infected with IBDV. At 48 h post-infection with IBDV, DF1 cells were collected for determination of IBDV VP2 gene expression levels. DF1 cells were transfected with either pEGFP-N1/CCL19 and sampled at 0, 4, 6, 8, 12, and 24 h post-transfection **(B)** or pEGFP-N1 control plasmid and sampled at 4, 6, 8, 12, and 24 h post-transfection **(C)**; then, p-JNK, JNK, and GAPDH expression were determined by western blotting. ** Indicates significant differences of relative fold changes on IBDV between pEGFP-N1 and other groups (*p* < 0.01).

As the above data indicated that CCL19 can decrease proliferative and survival capabilities of DF-1 cells and inhibit replication of IBDV in DF-1 cells, we next set out to investigate the molecular mechanisms underlying these effects. To screen the signal pathway by which CCL19 inhibited IBDV replication, p38, JNK, Akt, and Erk signal pathway inhibitors were administrated into DF1 cells before pEGFP-N1/CCL19 plasmid transfection. Figure 5A displayed relative IBDV VP2 gene expression levels for each group (p38 + CCL19, JNK + CCL19, Akt + CCL19, and Erk + CCL19) at 60 h after IBDV infection. When DF1 cells were treated with p38 and JNK signal pathway inhibitors, the inhibition effects of CCL19 on IBDV replication were offset, or even promoted IBDV replication, indicating that CCL19 could inhibit IBDV replication. Both Akt and Erk pathway inhibitors treatment had no effects on negative regulation of CCL19 on IBDV replication.

To further study why JNK pathway inhibitor treatment could promote IBDV replication even after CCL19 expression, pEGFP-N1-CCL19 plasmids were transfected into DF1 cells and JNK phosphorylation level were determined by western blotting on 4, 6, 8, 12, and 24 h after plasmids transfection. Obviously, pEGFP-N1-CCL19 could significantly elevate JNK phosphorylation level and activate JNK signal pathway, while the pEGFP-N1 control plasmids could not change JNK phosphorylation level (Figures 5B,C).

## Discussion

Similar to other chemokines, the functions of CCL19 mainly rely on immune cell trafficking. When CCL19 is mentioned, it is usually referred to the axis of CCR7/CCL19 and its effects on immune cell migration (Comerford et al., 2013). There are rare reports on the direct antiviral effects of CCL19. Herein, a eukaryotic expression vector pEGFP-N1/CCL19 was successfully constructed, and it could express in DF1 cells. More importantly, the plasmid pEGFP-N1/CCL19 was proved to inhibit IBDV replication *in vitro* at the gene level and protein level of IBDV. Moreover, the gene level of IBDV was recovered when CCL19 was knockdown by siRNA. The antiviral effects of CCL19 may be related to the activation of JNK signal pathway.

All the time, the main function of CCL19 is to control a serial of migratory events in immune responses, especially promoting T cells and DCs infiltration to microbial infection sites (Comerford et al., 2013). During the process of IBDV infection, one of the characteristics of IBDV pathogenesis is that with the depletion and inactivation of B cells in the bursa of Fabricius (Huang et al., 2021), numerous T cells are infiltrated into the target organ bursae of Fabricius after IBDV infection (Sharma et al., 2000). Our previous work has proved that CCL19 indeed played an important role in T cell migration during the process of IBDV infection (Wang et al., 2019). However, after RNA-seq was applied to analyze the transcriptional profiles of the responses of bursae of Fabricius in the early stage of IBDV infection, CCL19 was found that it could interact with a number of genes, such as regulator of G protein signaling 4 (RGS4), Annexin A1 (ANXA1), Bradykinin Receptor B2 (BDKRB2), Cytidine/Uridine Monophosphate Kinase 2 (CMPK2), and Eukaryotic Translation Initiation Factor 2 Alpha Kinase 2 (EIF2AK2) (Ou et al., 2017b). These results indicated that CCL19 not only took part in T cell migration during IBDV infection, but also might interact with other genes to affect IBDV pathogenesis. In this study, we justified our hypothesis that CCL19 could interfere the process of IBDV infection by inhibiting IBDV replication.

Avian T cell mediated immune responses were suspected to play a vital role in fighting virus infection, including IBDV, infectious bronchitis virus, avian influenza virus, and avian leukosis virus subgroup J (Seo and Webster, 2001; Wang et al., 2014; Tan et al., 2016; Xu et al., 2016). Indeed, T cell cytotoxic responses were activated by IBDV infection and could inhibit IBDV replication through Fas-FasL and perforin-granzyme pathways (Rauf et al., 2012). However, T cell exerted negative effects on an individual's health due to the delay in follicular recovery (Rautenschlein et al., 2002). As far as we know, almost no research has been directed toward the effects of T cell chemoattractant chemokine on IBDV replication. This is the first study to display that T cell chemoattractant chemokine CCL19 had positive effects on inhibiting IBDV replication besides the direct inhibition functions of T cell on IBDV replication.

Another interesting thing was that CCL19 could significantly inhibit the gene levels of VP1 and VP2, but not other three virus protein genes (data not shown). To further determine the effects of CCL19 on virus protein expression levels, monoclonal antibodies against VP2 and VP3 were acquired from a commercial company. As shown in Figure 3C, the antibody against VP2 was not so good and the target bands were very faint. There were always faint background dots on the blot. These results indicated that CCL19 could inhibit expression levels of VP2 and VP3 despite the limitations of primary antibodies. Moreover, when there was a knockdown of CCL19, IBDV VP1 and VP2 gene expression levels were increased obviously. These results indicated that CCL19 might influence the aspects of transcriptions and translations of IBDV. Of course, further studies on inhibition mechanisms of CCL19 on IBDV replication would be required.

Many signal pathways were involved with IBDV pathogenesis, including p38 MAPK pathway, JNK signaling pathway, and Akt signaling pathway (Khatri and Sharma, 2006; Wei et al., 2011; Zhang et al., 2020). Four important signaling pathway inhibitors for IBDV were chosen and administrated to find which pathway would take part in the process of CCL19 on IBDV inhibition in this study. First, the ERK pathway inhibitor did not influence the effects of CCL19 on IBDV replication, which indicated that ERK pathway did not interfere with the process of IBDV replication. To the authors' knowledge, there are no reports on the relationship of ERK pathway with IBDV replication. Interestingly, Akt pathway inhibitor could not stop the inhibition effects of CCL19 on IBDV replication. Host cell phosphatidylinositol 3-kinase (PI3K)/Akt signaling seems to be a sentinel pathway after virus infection and take part in a number of physiological changes in the process of virus infection, including IBDV growth (Wei et al., 2011), virus entry into cells (Ye et al., 2017), and inhibition of host cell autophagy (Zhang et al., 2020). Thus, the result that CCL19 still inhibited the IBDV replication after administration of Akt pathway inhibitor is uncanny. Our only reasonable explanation for this result is that CCL19 relies on another pathway to inhibit IBDV replication. After blockage of p38 pathway and JNK pathway, CCL19 did not inhibit IBDV replication. However, JNK pathway inhibitor promoted the IBDV growth. Therefore, JNK pathway plays an important role in the process of inhibiting IBDV growth by CCL19. Further study displayed that CCL19 activates JNK pathway. Actually, activation of CCL19 and its receptor CCR7 usually could induce JNK phosphorylation and promote cell migration (Liu et al., 2014), which further justifies our result.

To conclude, the T cell chemoattractant CCL19 possessed the ability of inhibiting IBDV replication other than recruiting T cell into IBDV target organ bursae. The antiviral effects of CCL19 might be related to the activation of JNK signal pathway. Further research will be also applied to explore the detailed mechanism of CCL19 on IBDV replication. Moreover, this study is only an *in vitro* study and whether CCL19 could be applied to control IBDV infection in poultry is still unknown. Antiviral roles of CCL19 and its administration time, frequency, and dosage form will be determined in an IBDV-infected chicken model.

## Data availability statement

The original contributions presented in the study are included in the article/supplementary material, further inquiries can be directed to the corresponding author/s.

## Author contributions

QW, FC, and XZ did experiment and wrote the manuscript. CO and XL designed the study and review the manuscript. HH, LL, FW, YY, YZ, JM, ZX, and FE took part in study design, data statistical analysis and reviewing. All authors contributed to the article and approved the submitted version.

## Funding

This study was supported by grant from the key Scientific and Technological Project of Henan Province (212102110009 and 202102110102), the Modern agricultural industrial technology system of Henan Province (S2012-06-G02), the Program for Innovative Research Team (in Science and Technology) in University of Henan Province (20IRTSTHN025), and the Key Scientific Research Project in University of Henan Province (21A230003).

## Conflict of interest

The authors declare that the research was conducted in the absence of any commercial or financial relationships that could be construed as a potential conflict of interest.

## Publisher's note

All claims expressed in this article are solely those of the authors and do not necessarily represent those of their affiliated organizations, or those of the publisher, the editors and the reviewers. Any product that may be evaluated in this article, or claim that may be made by its manufacturer, is not guaranteed or endorsed by the publisher.
