# Supplementary figures and images for: Infectious bursal disease virus replication is inhibited by avain T cell chemoattractant chemokine CCL19
Source: Front Microbiol. 2022 Jul 22;13:912908. doi: 10.3389/fmicb.2022.912908 (PMC9355407; doi:10.3389/fmicb.2022.912908)

Fig1. IBDV infection-48h

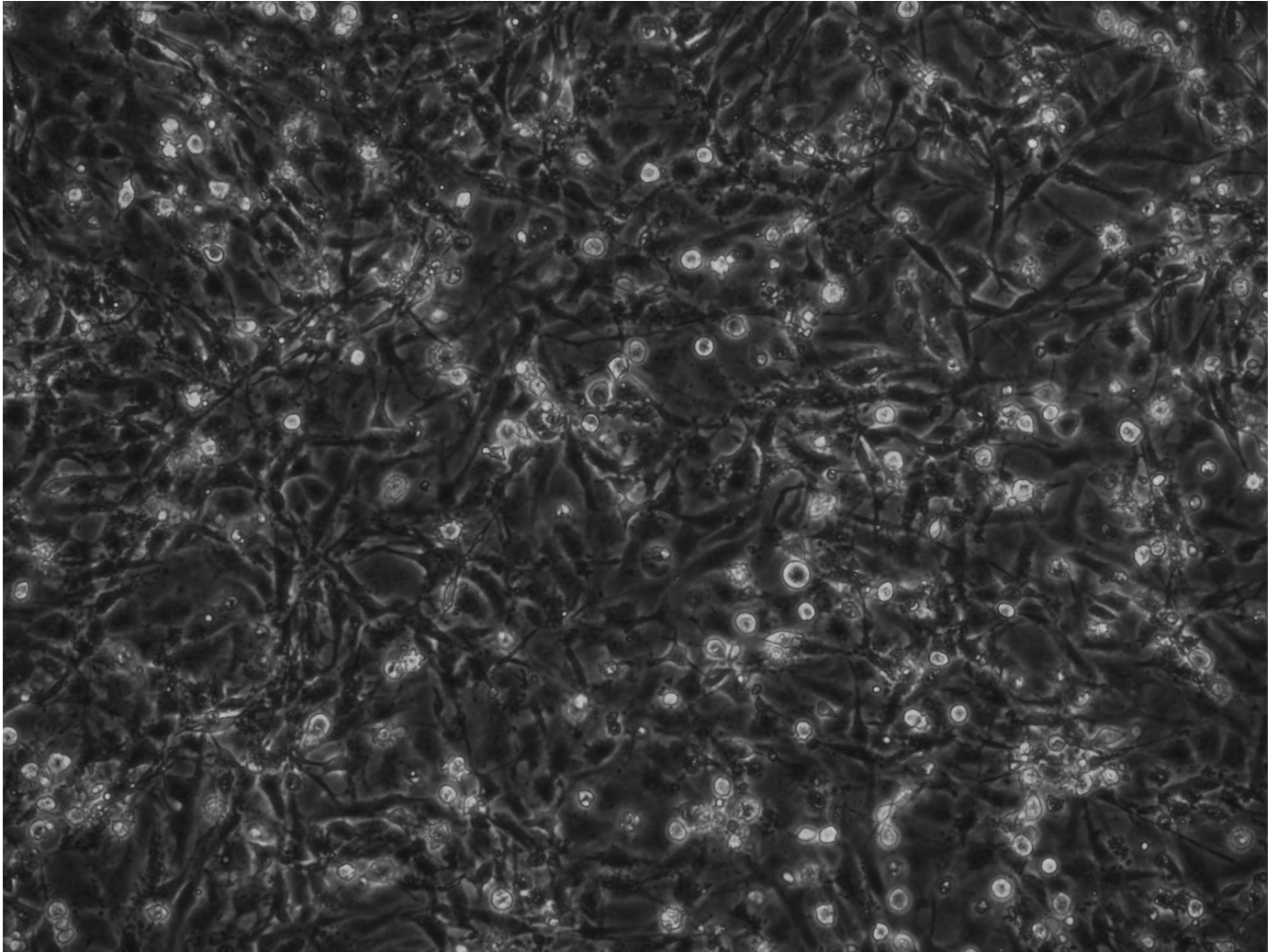

Fig1. IBDV infection-72h

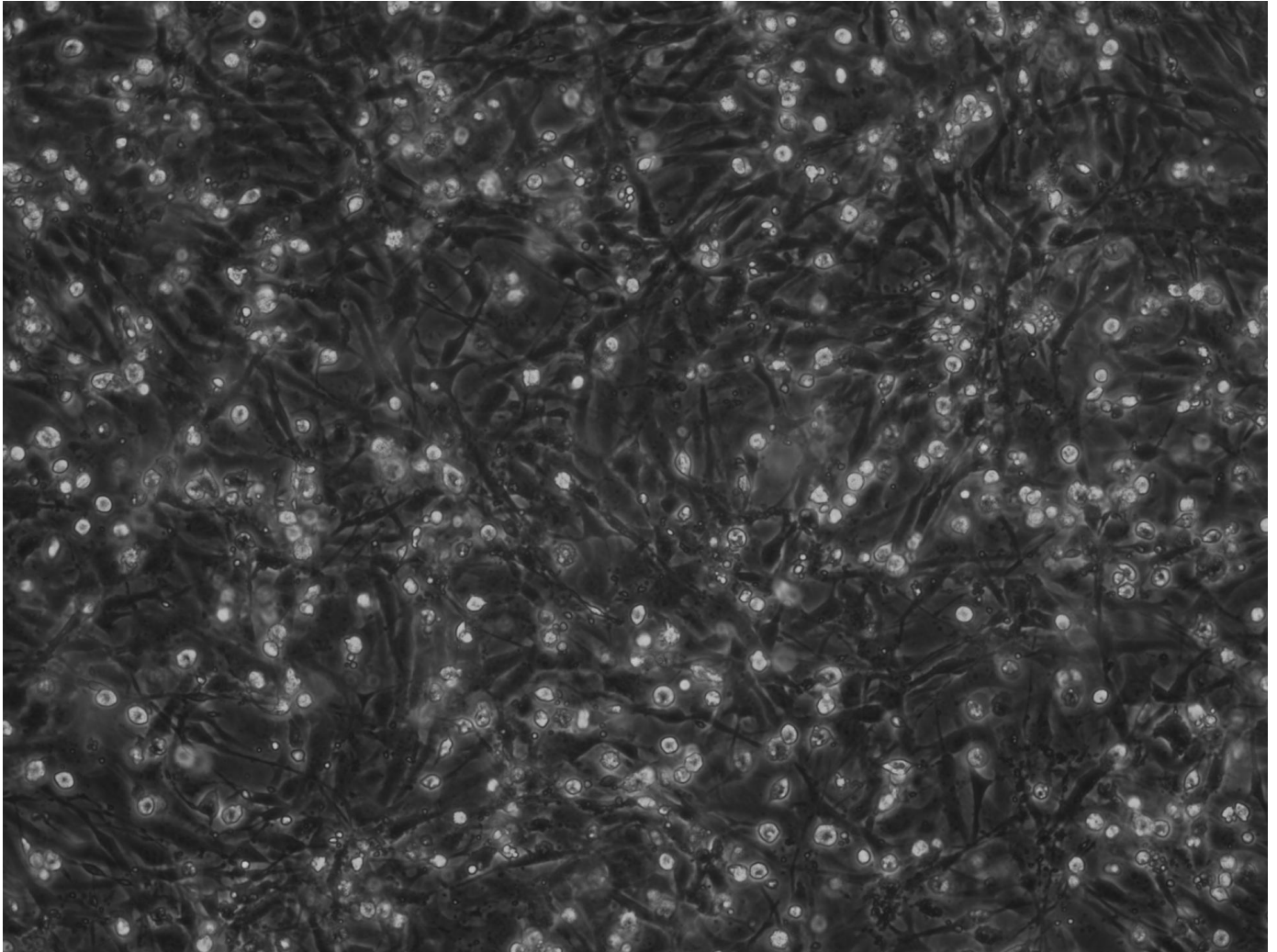

Fig1. IBDV infection-96h

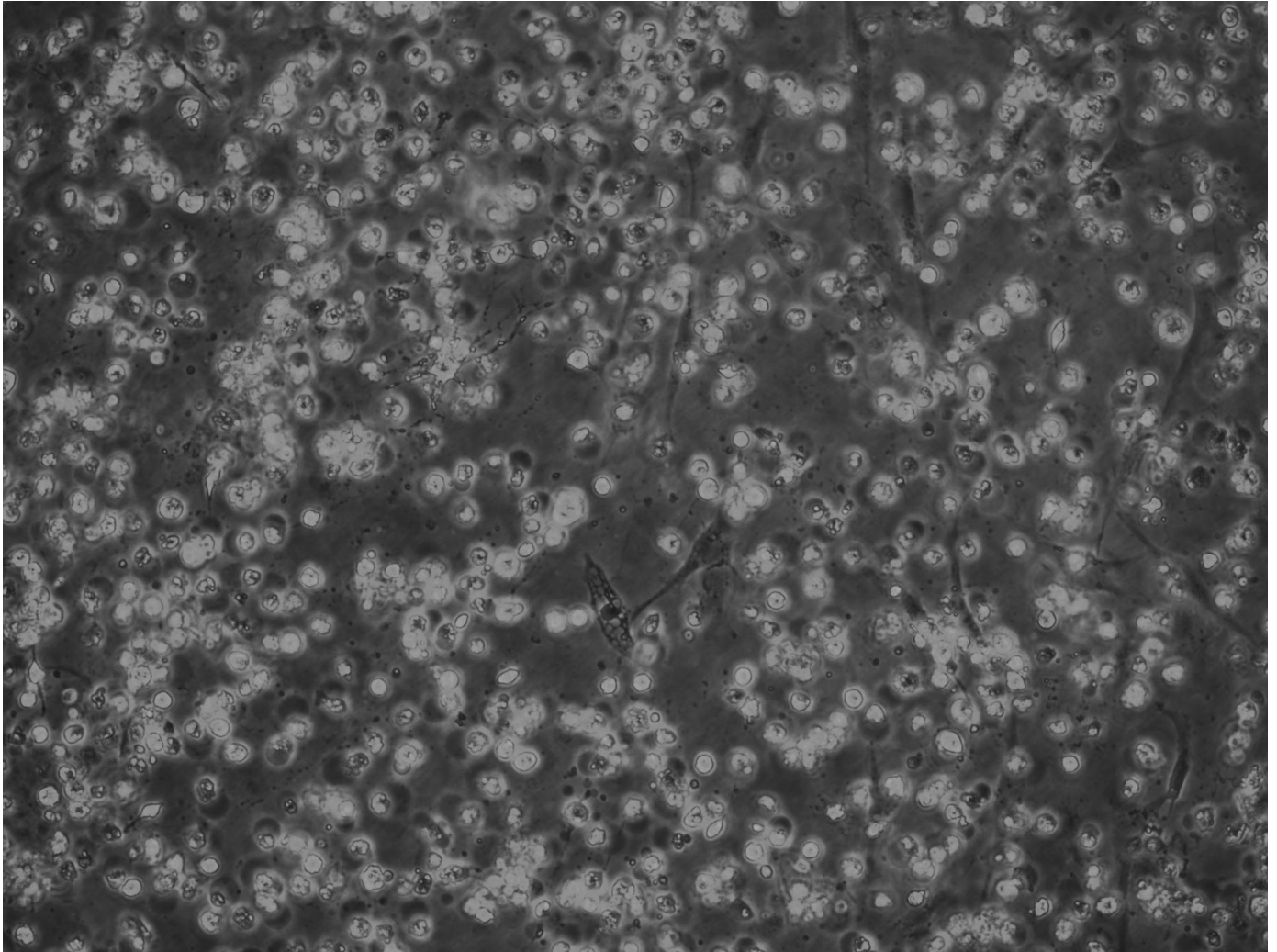

Fig.1 IBDV infection-24h

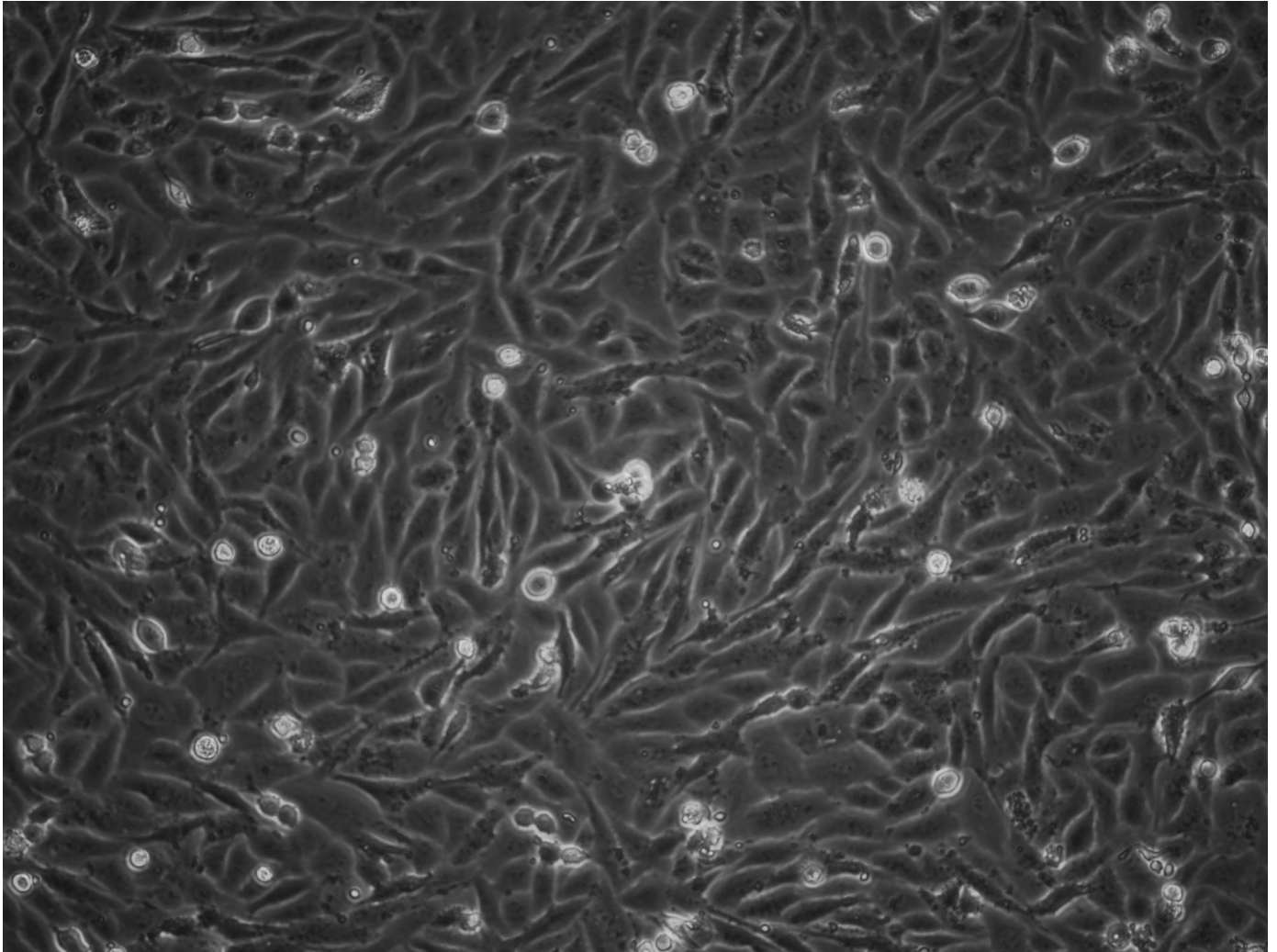

Supplement: Supplementary file 1 [file Data_Sheet_1.PDF]

Fig2. ccl 19 ( 0.5ug ) -12h

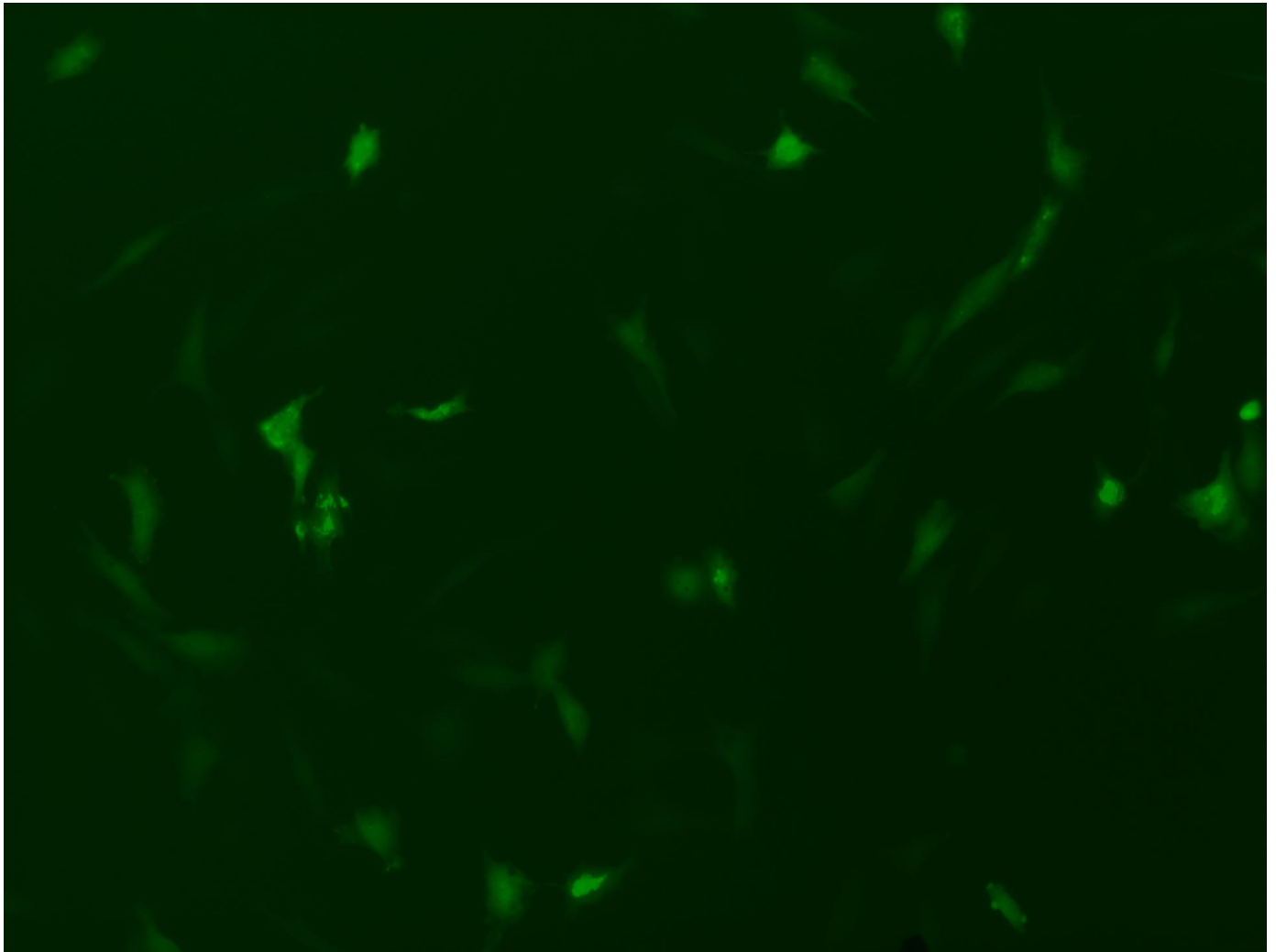

Fig2. ccl19 ( 1.5ug ) -12h

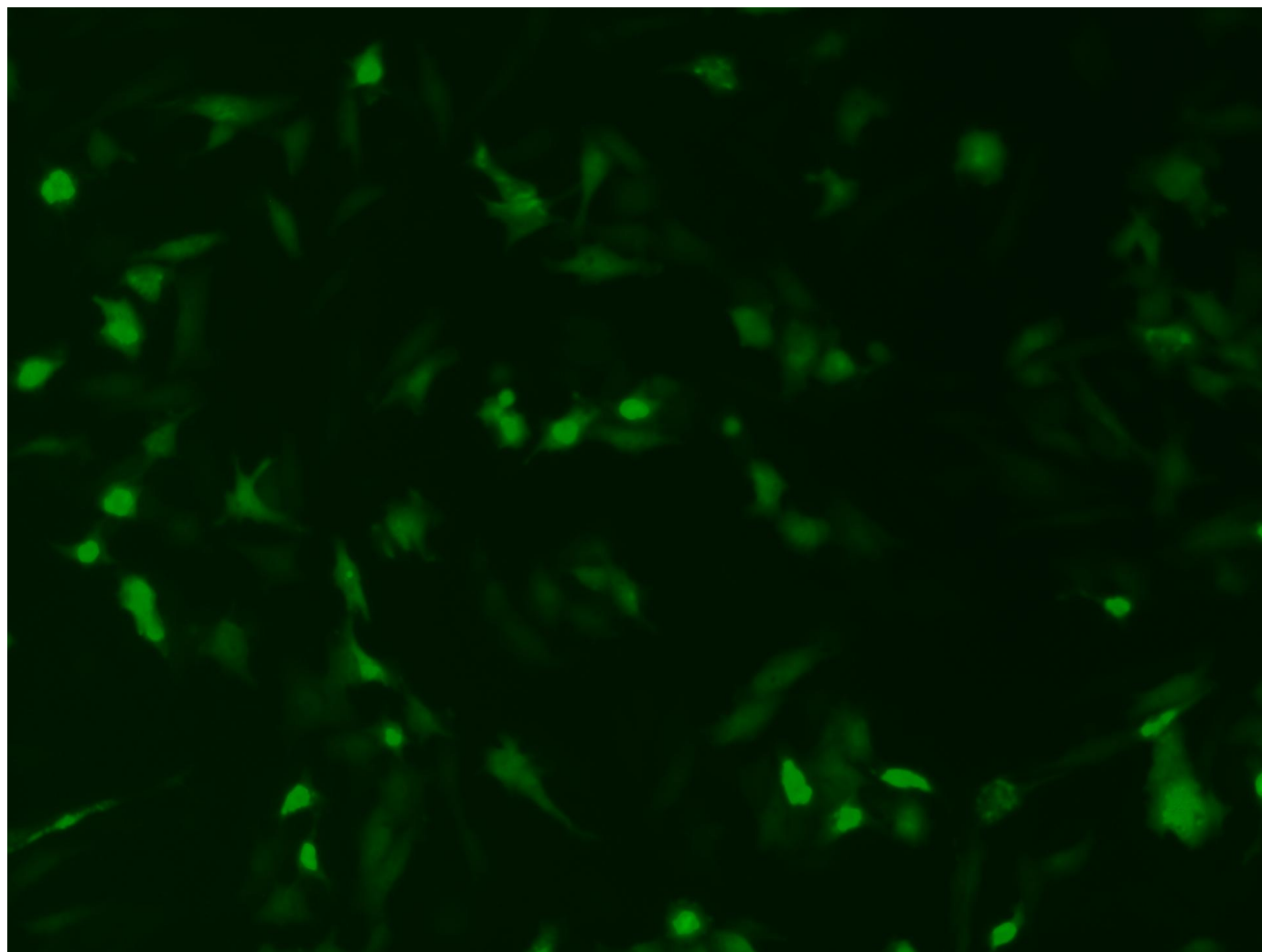

Fig2. ccl19 ( 1ug ) -12h

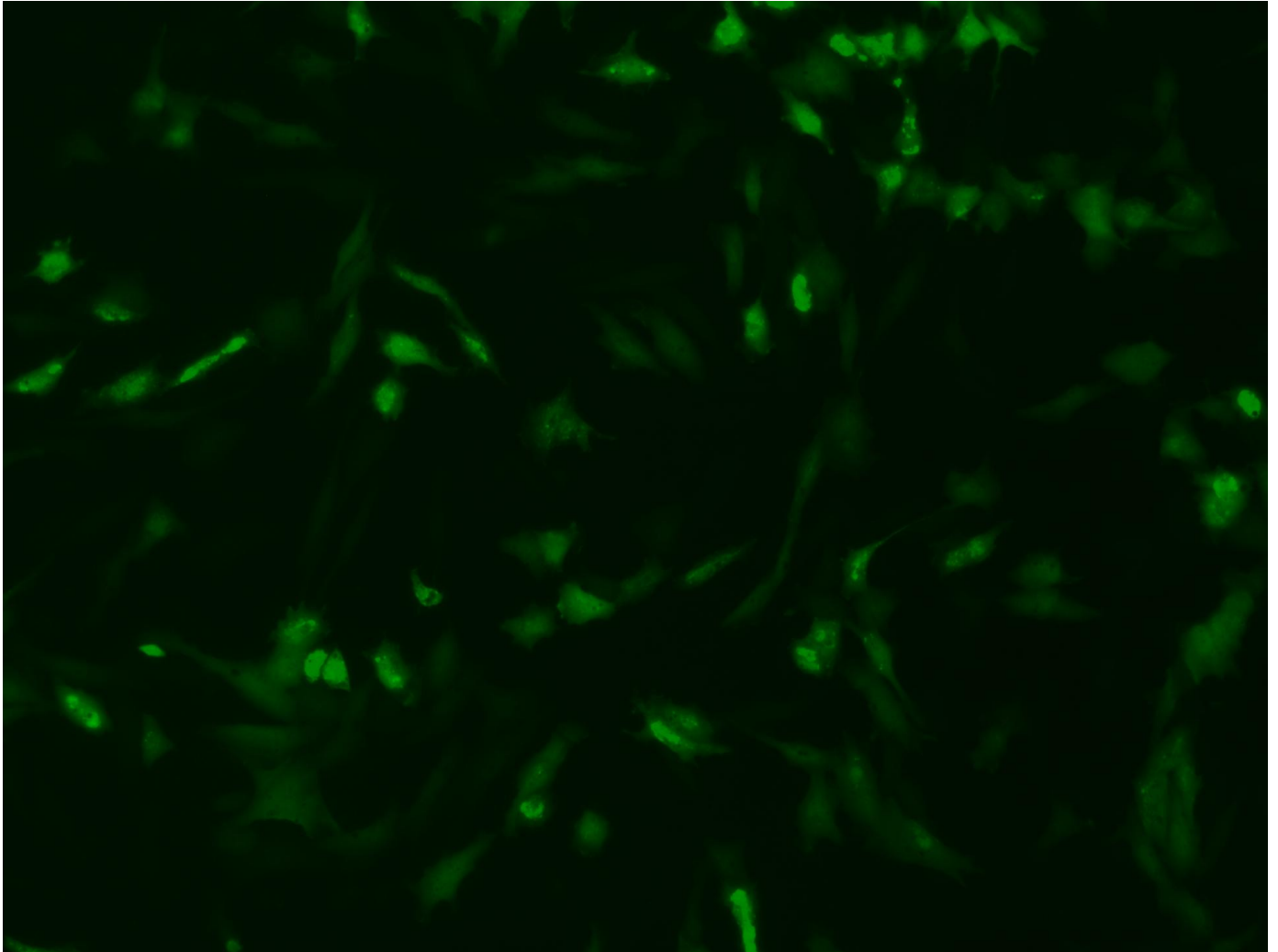

Fig2. ccl 19 ( 2ug ) -12h

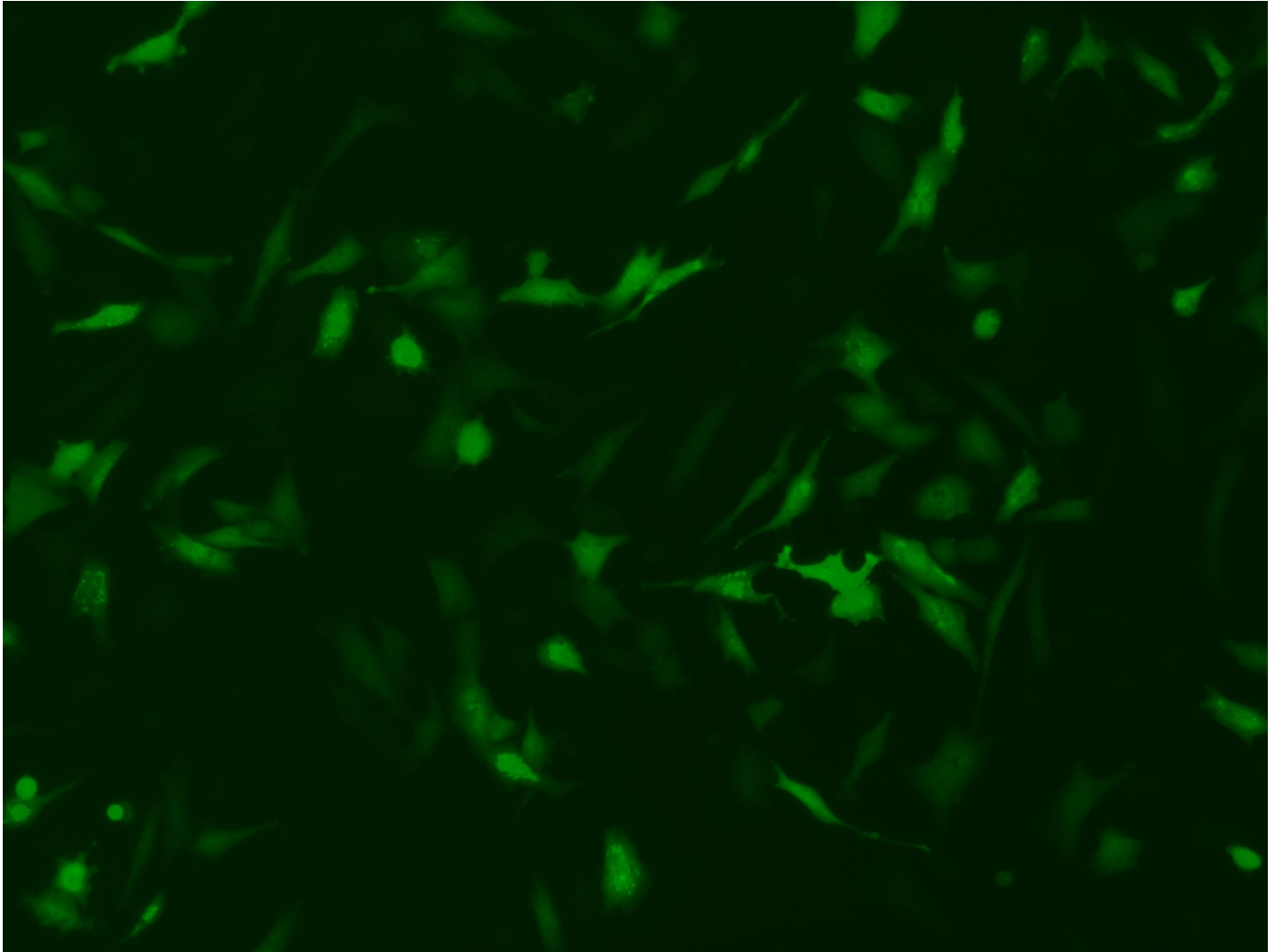

Supplement: Supplementary file 2 [file Data_Sheet_2.PDF]

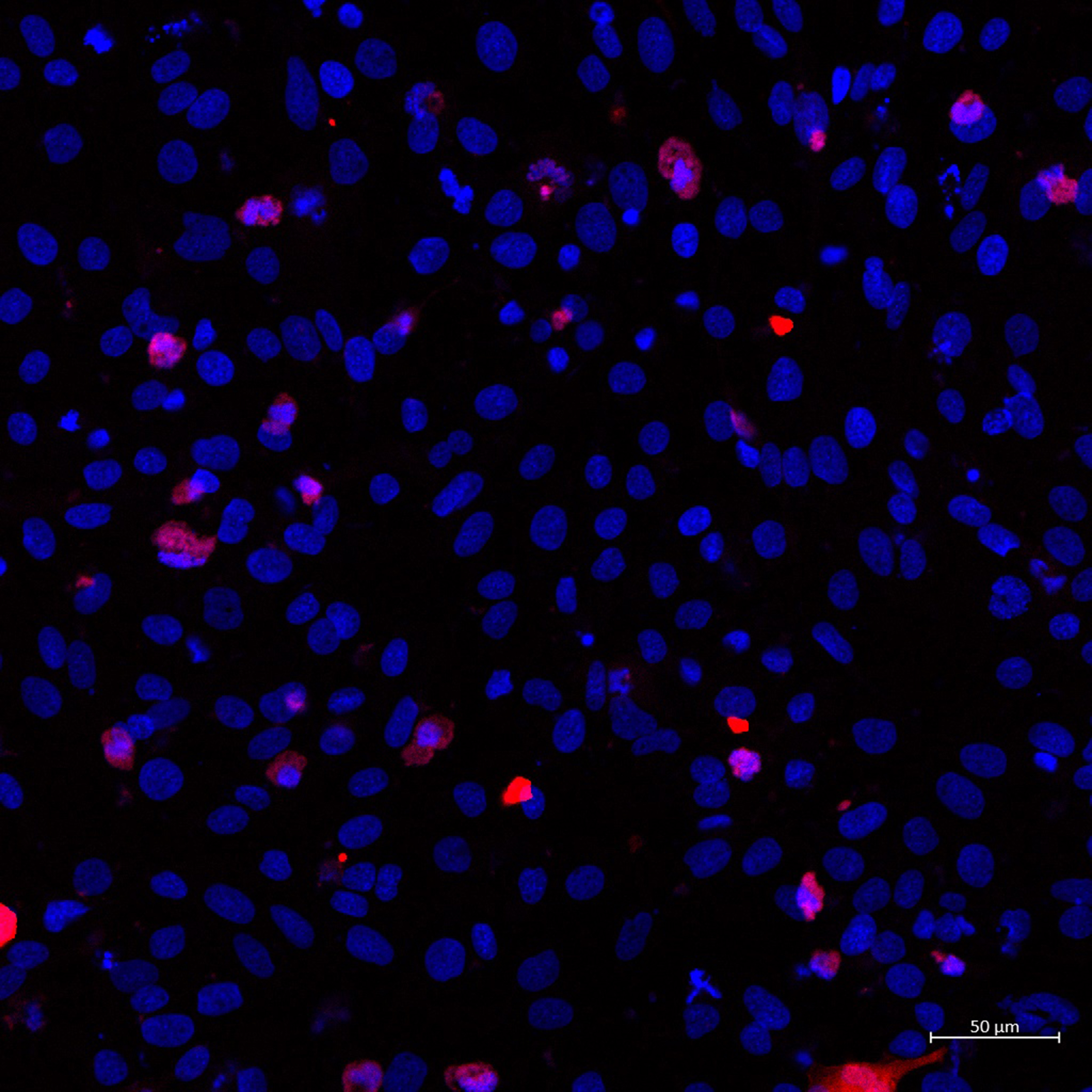

Supplement: Supplementary file 3 [file Image_1.JPG]

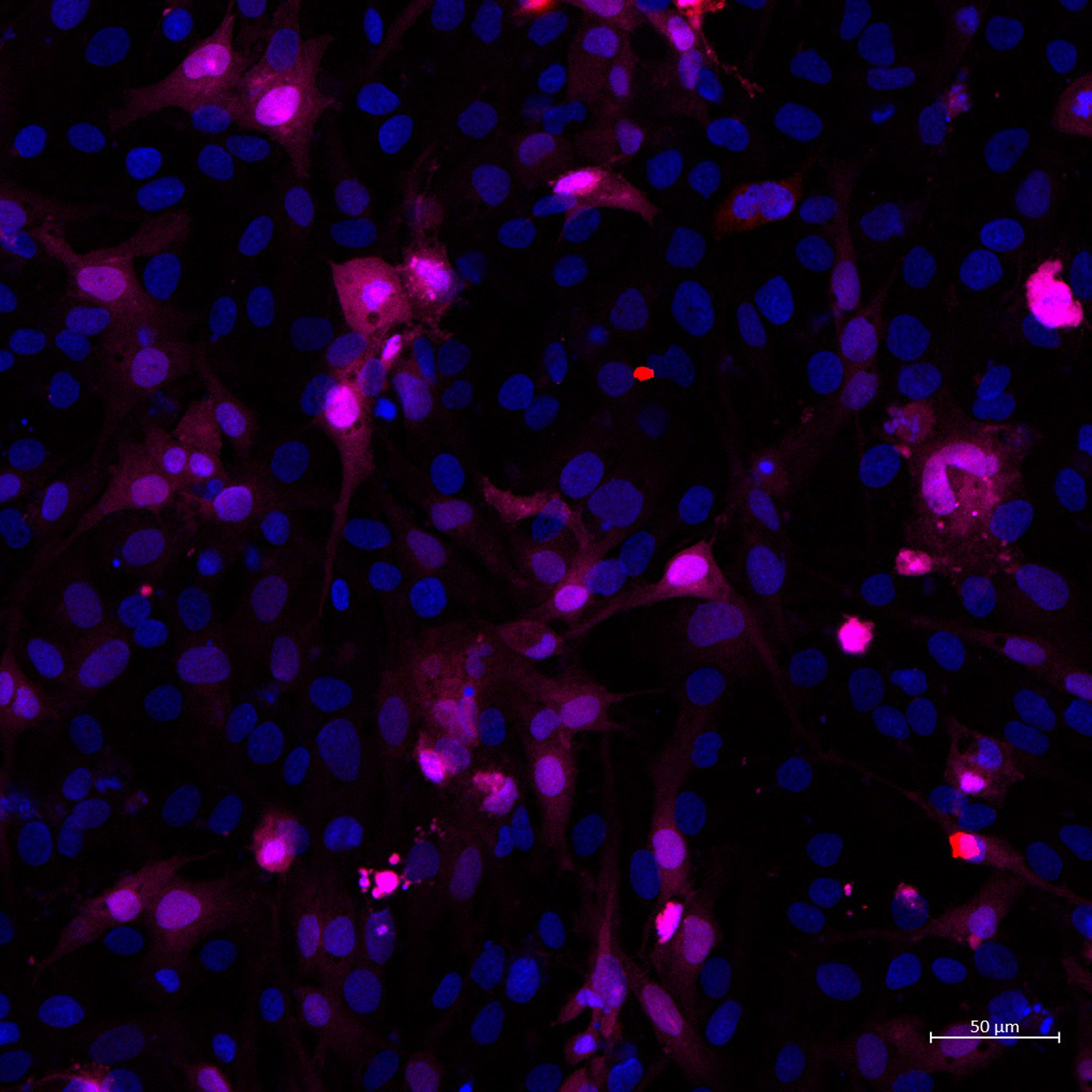

Supplement: Supplementary file 4 [file Image_2.JPG]

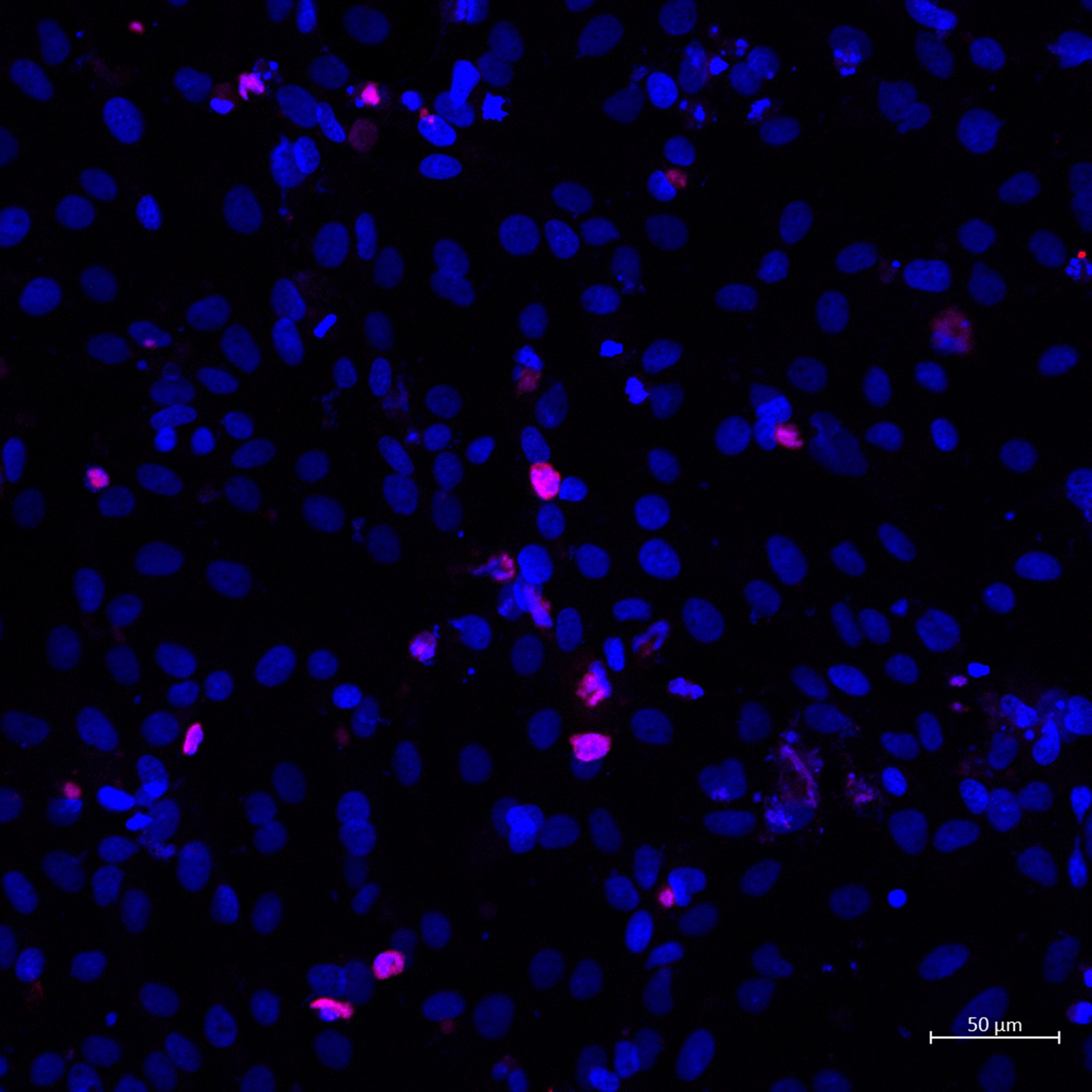

Supplement: Supplementary file 5 [file Image_3.JPG]

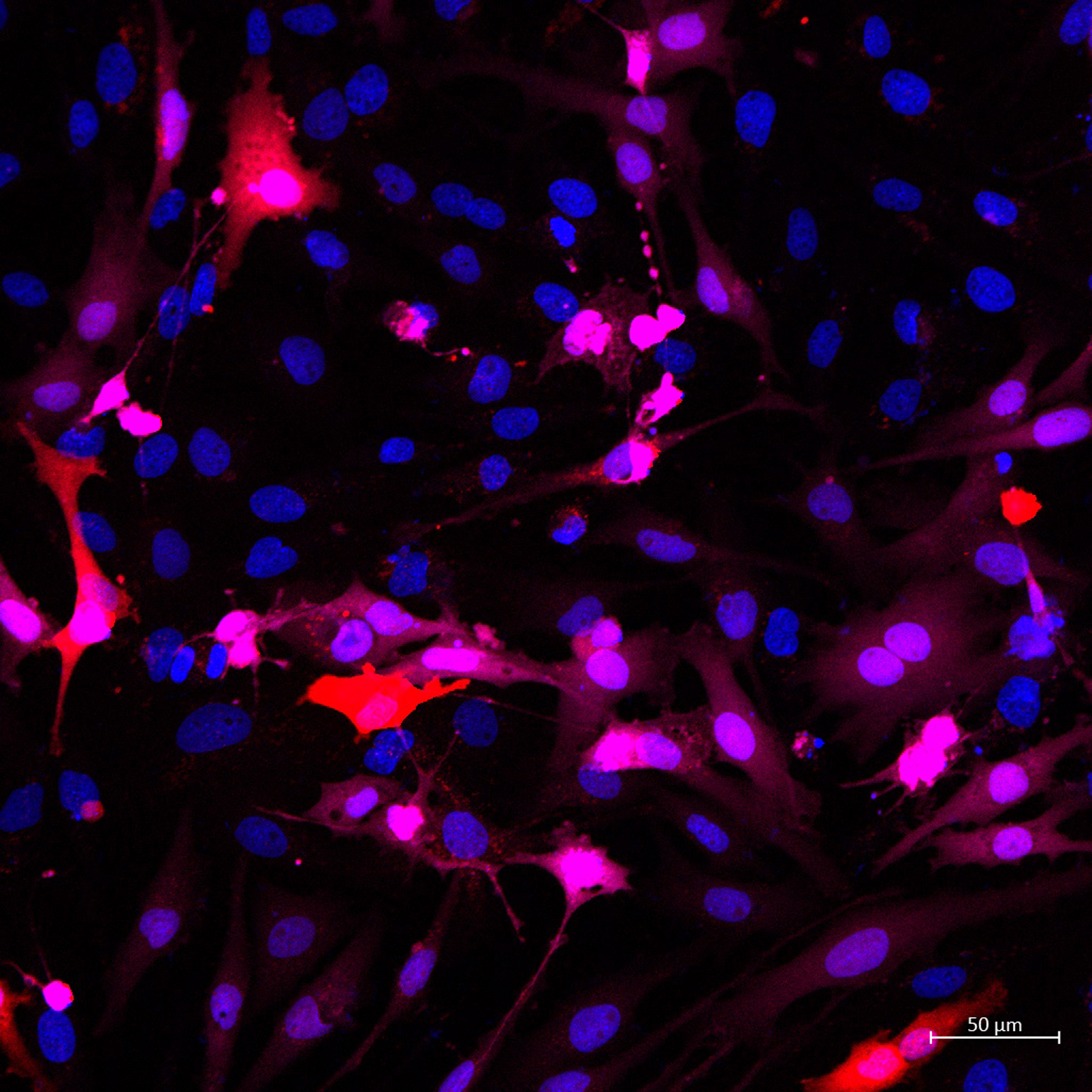

Supplement: Supplementary file 6 [file Image_4.JPG]

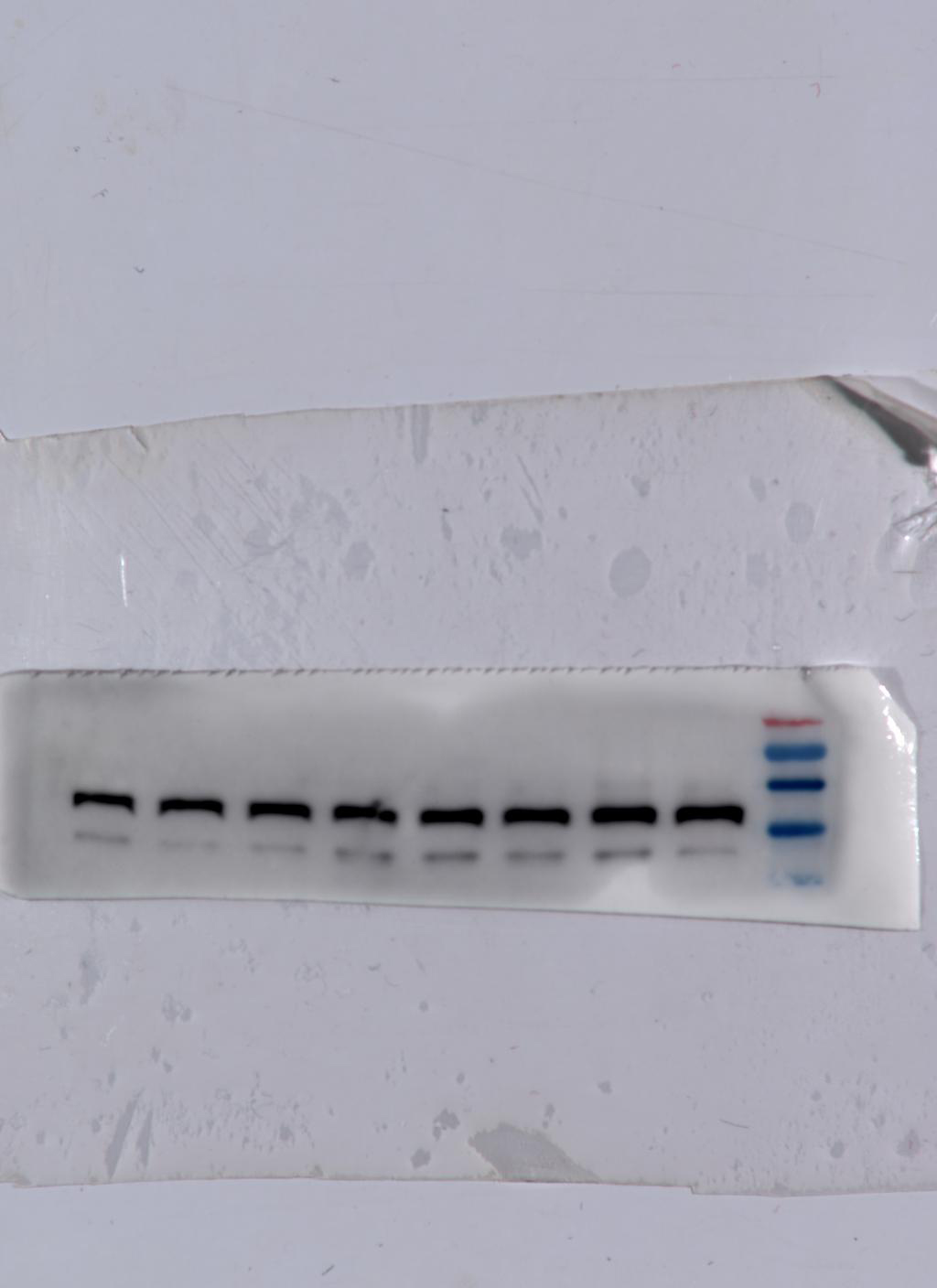

Supplement: Supplementary file 7 [file Image_5.JPG]

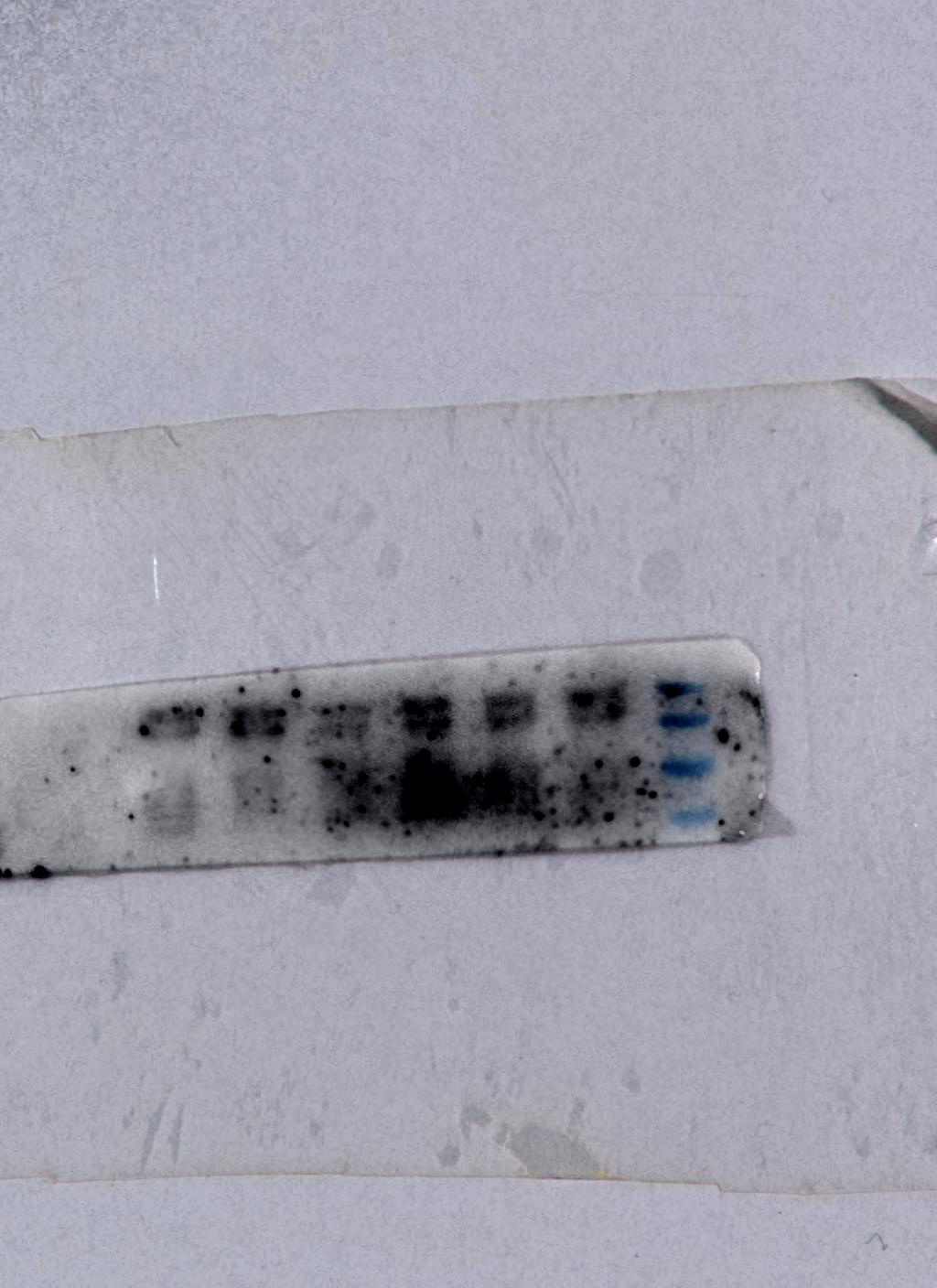

Supplement: Supplementary file 8 [file Image_6.JPG]

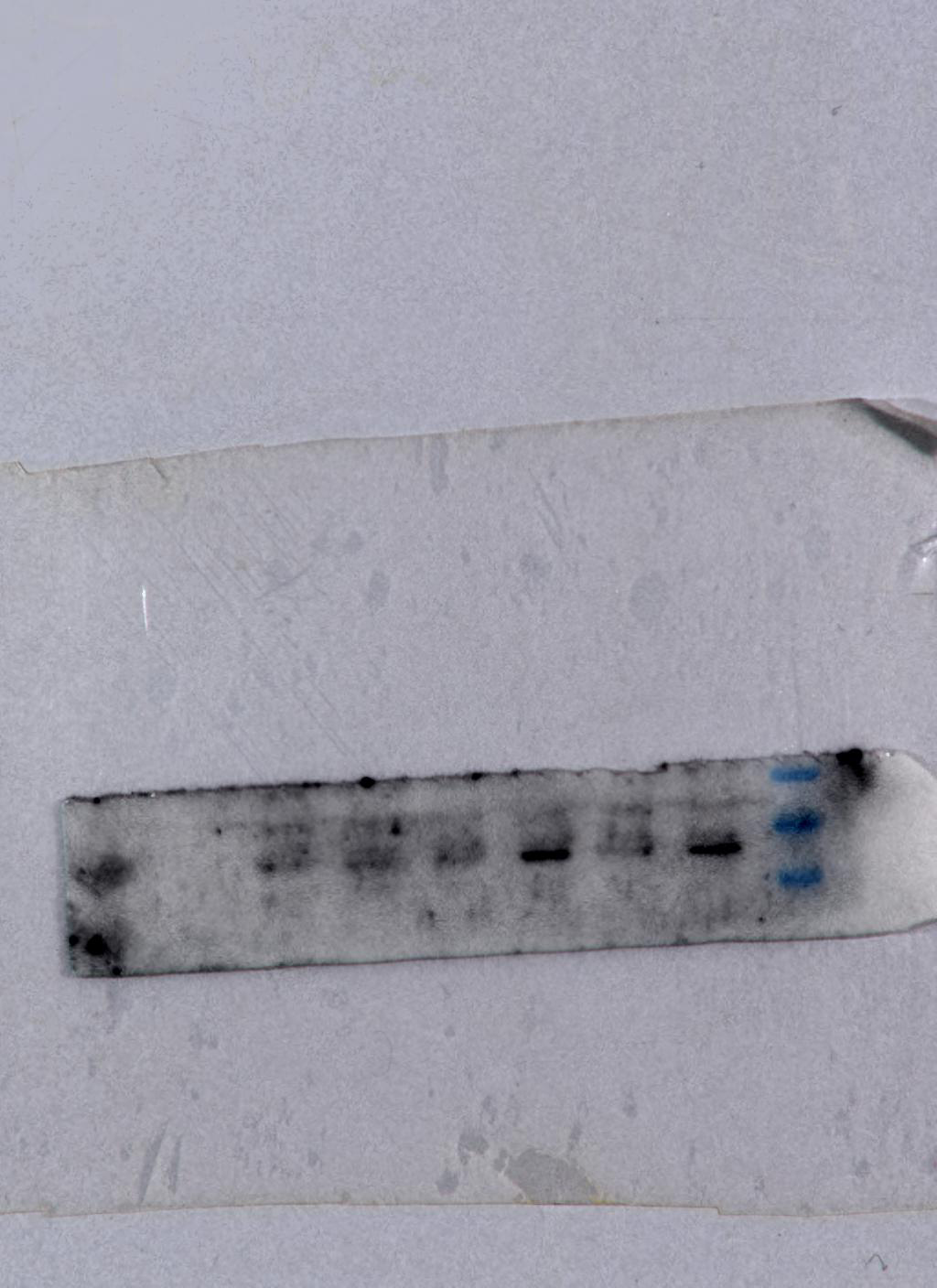

Supplement: Supplementary file 9 [file Image_7.JPG]

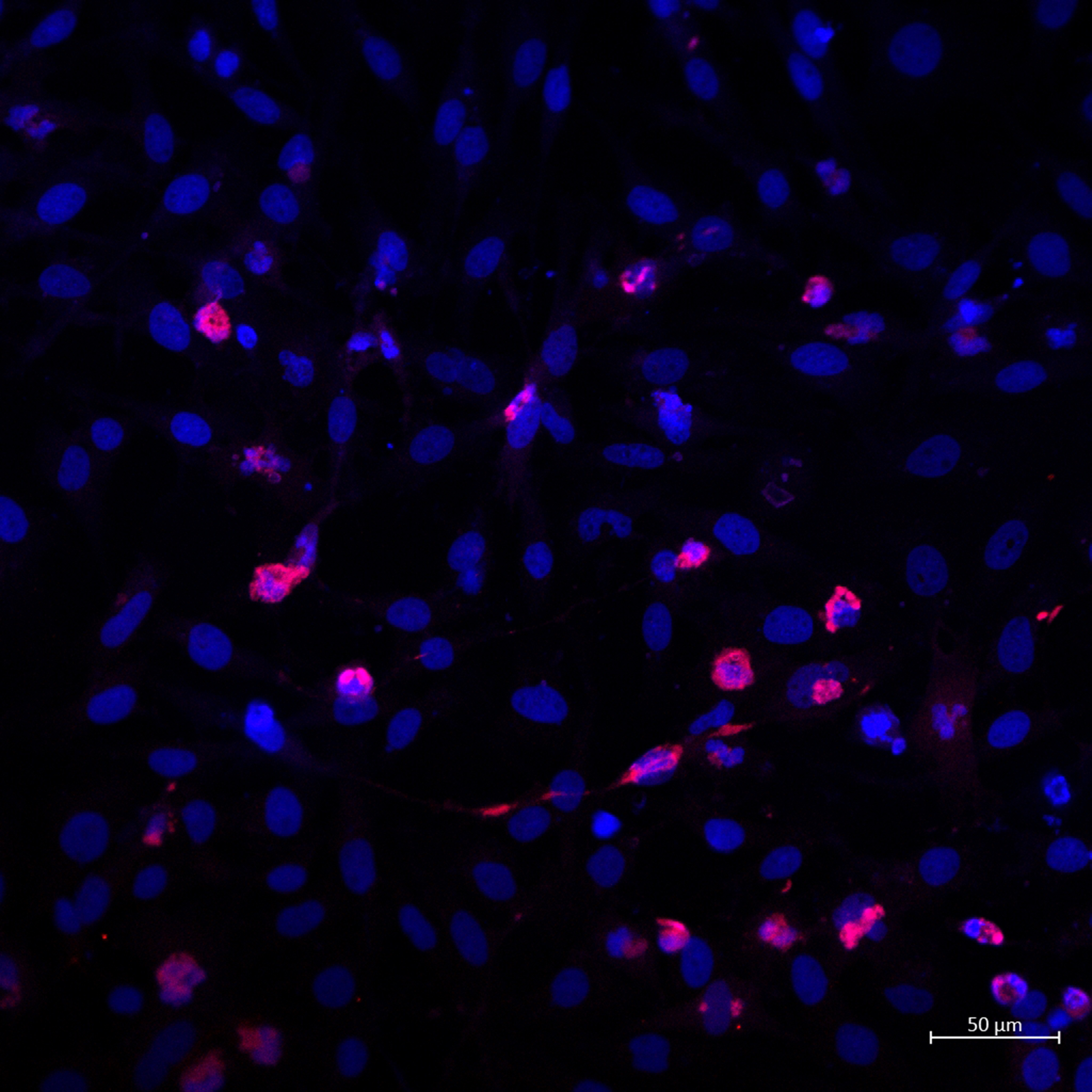

Supplement: Supplementary file 10 [file Image_8.JPG]

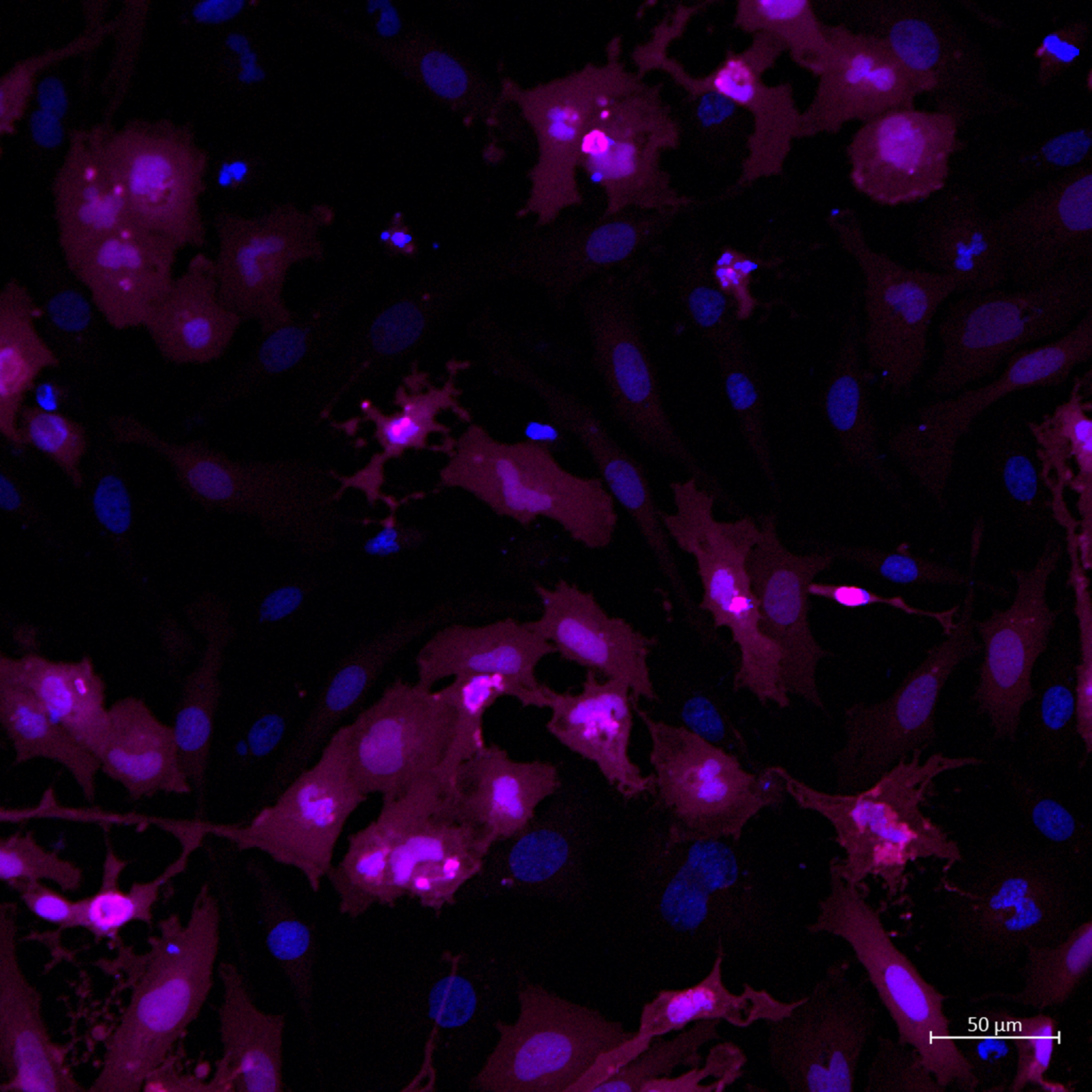

Supplement: Supplementary file 11 [file Image_9.JPG]
